# Molecular Complexes at Euchromatin, Heterochromatin and Centromeric Chromatin

**DOI:** 10.3390/ijms22136922

**Published:** 2021-06-28

**Authors:** Olivia Morrison, Jitendra Thakur

**Affiliations:** Department of Biology, Emory University, 1510 Clifton Rd #2006, Atlanta, GA 30322, USA; olivia.morrison@emory.edu

**Keywords:** histone modifications, CENP-A, H2AZ, epigenetics, compaction, transcription, silencing

## Abstract

Chromatin consists of a complex of DNA and histone proteins as its core components and plays an important role in both packaging DNA and regulating DNA metabolic pathways such as DNA replication, transcription, recombination, and chromosome segregation. Proper functioning of chromatin further involves a network of interactions among molecular complexes that modify chromatin structure and organization to affect the accessibility of DNA to transcription factors leading to the activation or repression of the transcription of target DNA loci. Based on its structure and compaction state, chromatin is categorized into euchromatin, heterochromatin, and centromeric chromatin. In this review, we discuss distinct chromatin factors and molecular complexes that constitute euchromatin—open chromatin structure associated with active transcription; heterochromatin—less accessible chromatin associated with silencing; centromeric chromatin—the site of spindle binding in chromosome segregation.

## 1. Introduction

Chromatin is a complex of DNA and histone proteins with many functions, including packaging genetic material to fit inside the cell and regulating gene expression. Walther Flemming first described chromatin around 1880 to describe the stainable threads he observed in the nucleus during mitosis [1]. Although the chemical composition of chromatin was discovered as DNA and protein, shortly after, the interest in chromatin structure only spiked after the discoveries that DNA was the “transforming principle” and that genes correlated with chromosomal bands [2,3,4]. In 1974, Kornberg proposed that the repeating unit of chromatin is made up of two copies of each of the four canonical histone proteins (H2A, H2B, H3, and H4) and approximately 200 bp of DNA [5]. Olins and Olins visualized chromatin structure as ‘beads on a string’ that were later termed as nucleosomes [4]. Among several models proposed for chromatin structure using low angle X-ray diffraction, the superhelical structure model garnered attention [6,7,8]. In 1997, the X-ray crystal structure of the nucleosome core particle at 2.8 A˚ revealed that 146 bp DNA is wrapped around the histone octamer in 1.65 turns of a left-handed superhelix [9].

Chromatin is divided into different categories based on the higher-order packaging of nucleosomes, histone post-translational modifications (PTMs) and histone variants [10,11,12]. Histone PTMs consist of the addition and removal of small chemical molecules such as methyl and acetyl groups to the histone tails [12]. Histone methylation is associated with the silencing of genes, while acetylation is associated with actively transcribing DNA by reducing the affinity of the histone octamer to DNA, rendering transcription easier [12,13,14,15]. Histone variants are the proteins that replace canonical histones from the nucleosomes at specific genomic locations and modify chromatin structures and functions. Common histone variants are H3.3 which replaces H3 in nucleosomes of active genes and Centromeric Protein A (CENP-A) found at centromeres [16]. 

The majority of chromatin is present as heterochromatin, a condensed chromatin structure that is transcriptionally less active and is mainly present at pericentric regions, telomeres, and other transcriptionally repressed regions. The second type of chromatin called euchromatin is more open, highly transcriptionally active and present in gene-rich genomic loci. The third specialized chromatin is present at centromeres where canonical histone H3 is replaced by its variant CENP-A. In addition to the core chromatin components, each type of chromatin is tightly associated and regulated by chromatin modifying proteins and protein complexes such as chromatin remodelers, histone-modifying enzymes, and architectural proteins that alter chromatin compaction [17,18,19,20,21,22]. Here in this review, we will discuss molecular complexes involved in the structural organization and function of euchromatin, heterochromatin and centromeric chromatin (Table 1).

## 2. Euchromatin

Euchromatin is characterized by active genes, wider spacing between nucleosomes, higher accessibility to transcription machinery, histone modifications and variants that facilitate active transcription [30,31,32,33,34,35,36]. The more open, unfolded structure of euchromatin allows transcriptional machinery to bind to the DNA, thereby facilitating its transcription. Euchromatin also contains the highest fraction of nucleosomes with exact nucleosomal positions relative to the underlying DNA sequence [37]. This nucleosome positioning is associated with the precise recognition of DNA sequence motifs by their protein partners and play an important role in transcription regulation near transcription initiation sites [38].

### 2.1. Genomic Regions Associated with Euchromatin

Euchromatin is mainly present on actively transcribing genes and regulatory elements such as promoters and enhancers, which regulate the access of transcription factors and chromatin remodelers to facilitate the opening of the chromatin and subsequent activation of transcription [31]. 

#### 2.1.1. Transcriptionally Active Gene Bodies

Transcriptionally active genes constitute a minor fraction of the genome and are usually located in the interior of the interphase nucleus where euchromatin is enriched. The gene body is the transcriptional region, including introns and exons, from the start site to the end of the transcript and is marked by activating histone modifications to help keep gene bodies in a more open chromatin state [39]. DNA methylation occurs on approximately 70–80% of the mammalian genome, but gene bodies are even more enriched for DNA methylation (80–90%) than intergenic sequences [40]. DNA methylation on gene bodies is believed to prevent intragenic transcription initiation in highly expressed genes [41,42,43]. Nucleosome positioning is weak on gene bodies as compared to the transcription initiation sites and further decreases gradually along the length of gene bodies [44].

#### 2.1.2. Promoters

Promoters are regions where transcription is initiated and are found typically upstream of or at the 5’ end of a protein-coding gene or non-coding genes. Promoters of RNA polymerase II (RNAPII) contain a core promoter, the minimal sequence required to properly initiate transcription, such as the TATA box and additional motifs [32]. The core promoter binds to general transcription factors (GTFs) such as TATA-binding protein, TFIIB, TFIIE, TFIIF, and TFIIH. These GTFs and RNAPII form the pre-initiation complex (PIC), which is sufficient to initiate transcription but with a lower basal activity [45]. Promoters are associated with activating histone modifications and contain a ∼150-bp nucleosome depleted region (NDR) adjacent to the transcription start site (TSS). However, recently NDRs have been shown to contain highly dynamic nucleosomes containing the histone variants H3.3 and H2A.Z [36,46]. NDRs are flanked by highly positioned nucleosomes [38]. Promoters are characterized by the presence of CpG islands, regions of GC-rich DNA that are found at approximately 70% of vertebrate promoters. Although the exact role of DNA methylation in transcription is not clear, the right amount of methylation at the promoter regions is required for proper gene expression and mice with reduced methylation levels resulting from lack of a particular DNA methyltransferase (DNMT) die early in development [47,48,49,50]. Additionally, Histone regulator A (HIRA) mediates histone H3.3 localization at active promoters and transcribed gene bodies [51].

#### 2.1.3. Enhancers

Enhancers are DNA sequences that activate transcription independent of their distance or orientation with respect to the promoters of genes to be transcribed by binding to a variety of transcription factors. Enhancers regulate transcription in a tissue-specific manner, thereby playing an important role in development and differentiation [52,53]. Enhancers transcribe bidirectionally into non-coding RNAs (ncRNAs) that are involved in regulating transcription [54]. The current model for long-range transcriptional regulation is that the distal enhancers are brought into physical proximity to their target promoters by chromatin looping mechanisms [55,56,57]. A recent study using super-resolution 3-dimensional (3D) fluorescence in situ hybridization and chromosome conformation capture revealed a decreased spatial proximity between the Sonic hedgehog gene and its enhancers during the differentiation of embryonic stem cells (ESCs) to neural progenitors, suggesting that models of enhancer–promoter communication need to encompass chromatin conformations other than looping [58]. Enhancers are associated with nucleosomes that are primarily modified by activating histone modifications and contain the histone variants H3.3 and H2A.Z, which are deposited in a replication-independent manner [36,59]. The nucleosomes that flank the transcription factor binding sites of enhancers are less mobile than the H3.3/H2A.Z hypermobile nucleosomes [60]. 

### 2.2. Histone Modifications Associated with Euchromatin

Euchromatic histone PTMs include lysine acetylation and methylation. While lysine acetylation is almost always associated with chromatin accessibility and transcriptional activity, methylation can have different effects depending on which amino acid residue is modified. Histone modifications have been shown to play an important role in many biological processes including memory formation and storage, the aging of tissues, and differentiation [61,62,63].

#### 2.2.1. H3K4me3

The trimethylation of histone 3 at lysine 4 (H3K4me3) PTM is a characteristic feature of chromatin present at promotor regions near TSS [64]. In mammals, H3K4 methylation is catalyzed by six related homologs of the yeast histone lysine methyltransferase SET1 (SETD1A, SETD1B, MLL1, MLL2, MLL3, and MLL4) [65]. SET1 associates with RNAPII and is deposited co-transcriptionally [66]. Promoters with low and high H3K4me3 levels are enriched for MLL2 and the CFP1 subunit of SETD1, respectively [67,68]. CFP1 has a PHD finger domain known to read H3K4me3 and mediate the interaction between SETD1 and H3K4me3 [69]. The third PHD domain in MLL1 is important for H3K4me3 binding and MLL1 recruitment to target sites in the Hox locus [70]. These mechanisms of H3K4me writers suggest that once established, H3K4me modification may positively reinforce its own deposition [12]. 

#### 2.2.2. H3K27ac

The acetylation of histone H3 at lysine 27 (H3K27ac) PTM is a well-recognized marker for active but not poised enhancers and is imparted by histone acetyltransferase (HAT) complexes [60]. However, H3K27ac alone does not functionally determine enhancer activity as substituting lysine 27 in histone variant H3.3 with arginine in mouse ESCs, does not perturb the transcription [71]. H3K27 acetylation is found concurrently with H3K4me3 at promoters and the TSS of active genes. H3K4me3 readers have been identified in many HAT complexes. For instance, SGF29, a component of the SAGA HAT complex, contains a domain that binds H3K4me3 and overlaps with H3K4me3 at gene promoters [72]. TSS-associated histone H3K27 acetylation signals the Super Elongation Complex (SEC) to regulate transcriptional elongation of the *ESR1* (*ERα*) gene by interacting with its scaffold protein AFF4, suggesting that the TSS H3K27ac is involved in bridging the transition from transcriptional initiation to elongation [73]. 

#### 2.2.3. H3K36me

Gene bodies of transcriptionally active genes are enriched mainly for activating methylation of histone H3 at lysine 36 (H3K36me3) PTM, which is imparted by SET2, the first H3K36 methyltransferase. SET2 is recruited for depositing H3K36me3 on gene bodies during transcription elongation by RNAPII phosphorylated at serine 2 [74]. SWT2 marks the H3K36 residue with mono-, di-, tri-methylation which are enriched over the 5′ ends, mid-coding regions, and the 3′ ends of genes, respectively, and each of these modifications has a distinct functional role but H3K36me1/2 and H3K36me3 have also been shown to act redundantly in many cellular contexts [34,75]. The H3K36me3 deposited by SET2 also serves as the binding site for histone deacetylase (HDAC) complexes to suppress excessive transcription initiation [76]. In yeast, the SET2-mediated H3K36me prevents hyperacetylation and histone exchange leading to well-spaced nucleosomes over the coding regions [33].

### 2.3. Histone Variants Associated with Euchromatin

Histone variants associated with euchromatic regions are found at regulatory elements such as enhancers and promoters as well as gene bodies. Histone variants play roles in cell fate decisions and development and are found to be deregulated in cancers at the transcriptional level [77].

#### 2.3.1. H2A.Z

H2A.Z is a replication-independent variant of canonical histone H2A. The C-terminal region of the yeast H2A.Z protein interacts with RNAPII, promoting its recruitment at promoters [30]. H2A.Z is recruited onto chromatin by the chromatin remodeling Swr1 complex, a member of the Snf2 family of ATPases [78]. The crystal structure of the central Swr1 enzyme in complex with the H2A.Z-H2B dimer revealed that Swr1 delivers the H2A.Z-H2B dimer to the nucleosome as a histone chaperone and partially unwraps the DNA from the histone core [20,79]. H2A.Z combined with H4 acetylation recruits Brd2, a double-bromodomain-containing protein known to function in transcriptional activation, to chromatin during transcription activation [35]. Furthermore, the acetyltransferase activity of the something about silencing (SAS) complex can stimulate H2A.Z incorporation in yeast, suggesting that histone acetylation facilitates H2A.Z deposition [80].

#### 2.3.2. H3.3

H3.3 is a conserved histone variant found at euchromatic actively transcribed regions and is incorporated into the nucleosome throughout the cell cycle, unlike canonical histones that are primarily deposited during replication [10,81,82,83]. H3.3 is enriched throughout the gene body of transcribed genes as well as at the promoter regions of both active and inactive genes that might be in their poised state [59,84]. In euchromatic regions, H3.3 deposition is mediated by the chaperone HIRA throughout the cell cycle [85]. However, H3.3 deposition also occurs at telomeres and pericentric heterochromatin, where it is catalyzed by the chaperone dimer, Atrx/Daxx [86,87]. A study using a H3.3K4A mutant has suggested that the Lysine 4 residue of histone H3.3 is required for ESC differentiation and transcription accuracy [88].

#### 2.3.3. H2A.B

The histone variant H2A.B (formerly named H2A.Bbd in humans) binds preferentially to H4 hyperacetylated regions and was named Barr body-deficient due to its exclusion from the inactive female X chromosome (Xi) [89,90]. When ectopically expressed in HeLa cells, H2A.Bbd is enriched on active genes and is involved in transcriptional activation and mRNA processing [91,92,93]. Factors involved in DNA replication are enriched in GFP-H2A.Bbd containing nucleosomal arrays in an asynchronous population of cells [94]. GFP-H2A.Bbd localization strongly correlates with sites of DNA replication, suggesting that H2A.Bbd is targeted to newly synthesized DNA during replication and repair [94]. H2A.B has been recently identified as a novel parental effect gene, where the *H2A.B* status of both the father and mother, but not the zygote, affects embryonic viability and growth during gestation [95].

### 2.4. Histone Chaperones

**Figure 1:**
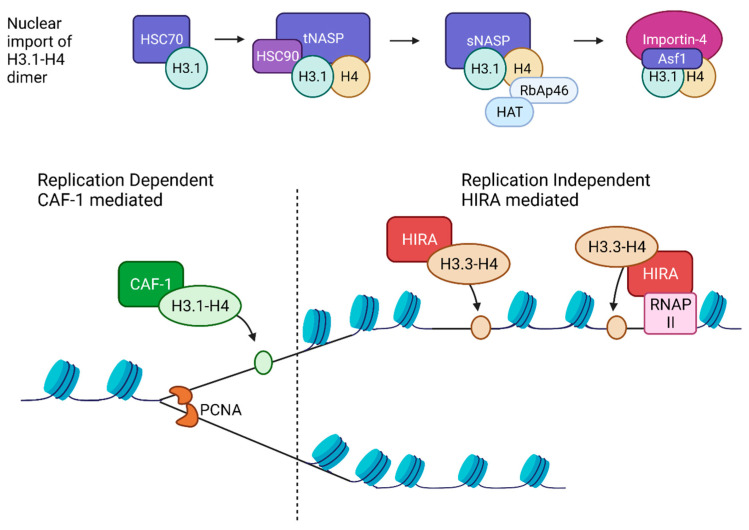
Nuclear import and deposition of newly synthesized H3-H4 molecules by histone chaperones into nucleosomes. Newly synthesized H3.1 associates with different chaperones in the process of nuclear import. The H3.1-H4 dimer is then assembled into a nucleosome in a replication-dependent method by its chaperone CAF-1, which is being recruited by proliferating cell nuclear antigen (PCNA) at replicating DNA. Newly synthesized H3.3-H4 is deposited onto DNA by the HIRA chaperone.

Histone chaperones are a group of proteins that bind to soluble histones and regulate nucleosome assembly. Histone chaperones can be categorized based on their assembly mechanism, either as replication-dependent (replication coupled) or replication-independent (Figure 1). Following synthesis, new H3-H4 molecules form distinct complexes with different histone chaperones to regulate free histone levels and nuclear import, likely impacting the deposition of new H3-H4 onto DNA.

#### 2.4.1. Replication-Dependent Nucleosome Assembly

Coupling of DNA replication to nucleosome assembly was established early on [96,97]. Subsequently, by studying lagging-strand synthesis, Okazaki fragments were found to be similar in size to the nucleosomal repeat length and the deletion of a subunit of histone chaperone CAF-1 involved in replication-coupled nucleosome assembly caused Okazaki fragments to increase in length [98]. Newly synthesized H3.1 associates with the protein chaperone Hsc70 before being assembled into a larger complex containing histone chaperone t-NASP, histone H4, and protein chaperone Hsp90 [99]. Then, H3-H4 associates with Hat1-RbAp46, a lysine acetyltransferase, Asf1 histone chaperone, and importin-4 [99]. Asf1 bound new H3-H4 is imported into the nucleus and assembled into nucleosomes by other chaperones. Although both H3 and H4 were considered to be imported into the nucleus as heterodimers, using a tether-and-release system and immunofluorescence, H3 and H4 were detected alone without binding partners, suggesting they may exist in the cytoplasm as monomers [100]. CAF-1 is a central replication-dependent chaperone and primarily functions to deposit newly synthesized H3-H4 onto newly synthesized DNA [101]. CAF-1 is recruited to the replication fork by proliferating cell nuclear antigen (PCNA), where it receives H3-H4 dimers from Asf1 and deposits them onto DNA to form a tetrasome, initiating nucleosome assembly [102].

#### 2.4.2. Replication Independent Nucleosome Assembly

Nucleosomes pose as barriers for transcriptional machinery and therefore they must be remodeled and reassembled following transcription through DNA replication-independent assembly [103]. Replication-independent nucleosome assembly is also the predominant way of histone replacement in non-dividing cells [104]. Many histone chaperones mediate replication-independent assembly of H3.3-H4 into nucleosomes. HIRA is the canonical histone chaperone involved in the replication-independent assembly of H3.3-H4 [85]. It has been shown that cells lacking HIRA do not have any effect on the localization of H3.3 to telomeres or regulatory elements, but H3.3 localization is reduced at gene bodies, thus suggesting HIRA is required for assembly and exchange of H3.3 at genic regions [59]. Daxx, another H3.3 histone chaperone, forms a complex with ATRX and a lack of ATRX leads to defects in H3.3 at telomeres and pericentric regions [59,105]. 

After H3-H4 tetramer deposition, H2A-H2B dimers are incorporated to complete the nucleosome with the help of chaperone Nap1 [106]. While H3-H4 tetramer is relatively stable, H2A-H2B dimers rapidly exchange with free H2A-H2B [107,108]. Nap1 regulates nucleosome assembly by facilitating H2A-H2B import, disrupting non-productive histone–DNA interactions, and depositing histones onto DNA. Nap1 facilitates histone import by mediating the interactions between KAP114 and H2A-H2B [106]. Nap1 promotes nucleosome formation by disassembling non-productive histone interactions [109]. Nap1 acts with ATP-dependent chromatin and remodeling factor complex to assemble regularly spaced nucleosomes and deposits both H3-H4 and H2A-H2B for nucleosome formation [110,111]. Another histone chaperone, FACT (facilitates chromatin transcription) binds to both oligomers of H2A-H2B and H3-H4. FACT binds H2A-H2B dimers by interactions with Spt16 MD and the CTDs of those subunits [112,113]. Partial DNA unwinding has been proposed to allow for FACT to bind both H2A-H2B dimers simultaneously during reorganization [113,114]. 

### 2.5. Chromatin Remodelers

**Figure 2:**
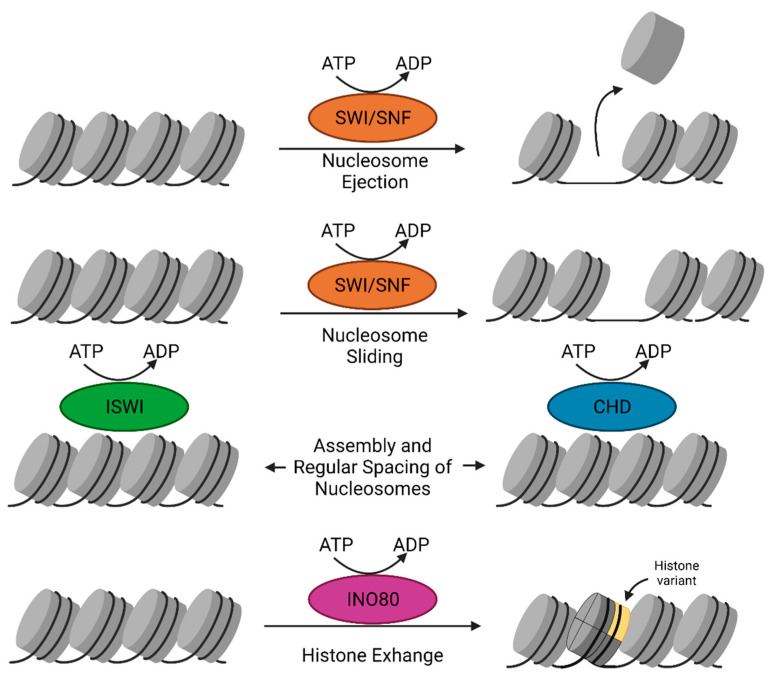
ATP-dependent chromatin remodelers. The SWI/SNF family of remodelers eject and slide nucleosomes to facilitate chromatin access. ISWI and CHD family remodelers assemble and regularly space nucleosomes. The INO80 family remodelers can have unique functions but are known to partake in histone variant exchange.

Chromatin remodelers catalyze a variety of nucleosome restructuring reactions, including nucleosome sliding, conformation changes of nucleosomal DNA, and histone variant exchange, that play a role in chromatin accessibility and are necessary for all DNA-dependent biological processes [115]. Chromatin remodelers contain an ATP hydrolyzing-DNA translocase unit that performs nucleosomal DNA translocation to disrupt the contacts between histones and DNA by utilizing energy from ATP hydrolysis. ATP-dependent chromatin remodelers are classified into four families that perform distinct modes of remodeling: switch/sucrose-non-fermenting (SWI/SNF), imitation switch (ISWI), chromodomain-helicase-DNA binding (CHD), and inositol requiring 80 (INO80) (Figure 2). Besides a common ATPase domain, chromatin remodeler families contain specific domains, for example bromodomain in SWI/SNF remodelers, HAND-SANT-SLIDE (HSS) molecules in ISWI remodelers, tandem chromodomains in CHD remodelers and finally, HAS domains in INO80 remodelers [116]. 

#### 2.5.1. SWI/SNF Complex

The first ATP-dependent chromatin remodeler described was the yeast SWI/SNF complex. The complex is targeted to acetylated histones through the bromodomain subunit [117]. The SWI/SNF complex also contains actin and/or actin-related proteins (ARPs) at its catalytic core [21]. The SWI/SNF complex is mostly involved in gene transcriptional activation [118]. The SWI/SNF family of remodelers typically facilitate chromatin access as they slide and eject nucleosome, and thus, they can be used for either gene activation or gene repression [119].

#### 2.5.2. The ISWI Family

The ISWI family of remodelers contains an ATPase domain containing two RecA-like lobes separated by a small insertion sequence [120]. They also contain a carboxy-terminal HSS domain that binds the unmodified histone H3 tail and the linker DNA flanking the nucleosome [121,122,123]. Most ISWI subfamily complexes assemble and regularly space nucleosomes to limit chromatin accessibility and gene expression. However, a subset of the ISWI family, such as the nucleosome remodeling factor (NURF) complex, has other subunits that result in activation of transcription [124,125].

#### 2.5.3. The CHD Remodeler Family

The CHD remodeler family is unique due to the presence of tandemly arranged chromodomains [126,127]. CHD remodelers take part in several processes in metazoans, including nucleosome assembly/spacing, providing access to promoters, and incorporation of histone H3.3 [126,128,129]. The nucleosome remodeling deacetylase (NuRD) complex helps repressors bind to chromatin and repress genes through its deacetylases [128,130]. In contrast to this, yeast Chd1 acts as a monomeric remodeler and mainly functions in chromatin assembly [127]. 

#### 2.5.4. The INO80 Subfamily

The INO80 subfamily of remodelers contains a large insertion between its RecA-like lobes. The N terminus of INO80 remodeler ATPases contains a HAS domain that nucleates actin and ARPs [131]. The INO80 subfamily contains various subtypes that have unique functions. The subtype INO80C moderates chromatin access and nucleosome spacing, while subtypes SWR1C, p400, and Snf2-related CBP activator protein (SRCAP) complex replace canonical H2A-H2B dimers with H2A.Z histone variant dimers [78,132]. Additionally, the p400 subtype has been shown to replace H3.1 with H3.3 [133].

### 2.6. Role of ncRNA in Euchromatin Organization

A significant fraction of nuclear RNA associates with chromatin and has been suggested to plan an architectural role in chromatin structure [134,135,136,137]. Besides structural RNAs, a large number of euchromatic proteins also bind to regulatory long ncRNAs (lncRNAs) that originate from loci in trans, suggesting a role for RNA in holding together two or more distant sites [138,139,140,141]. NEAT1 and MATAL1 lncRNAs are components of paraspeckles and nuclear speckles, respectively, and interact with transcripts from several euchromatic loci [141]. Enhancer derived ncRNAs are also involved in chromatin interactions such as looping, but it is unclear whether or not the effect is due to the RNA or just a consequence of enhancer activation [138,142,143].

Transcription of the male X chromosome in *Drosophila* is doubled to ensure equal levels of X-linked genes in males and females. This dosage compensation process is carried out by the dosage compensation complex (DCC, or MSL complex). Upregulation of the X chromosome transcription is partly due to a key component of DCC, the MOF (males absent on the first), which hyperacetylates histone H4 at lysine 16 [144]. Two noncoding RNAs exist in the DCC, *roX1* and *roX2,* that, while functionally redundant, loss of both dramatically affects male viability [144]. The DCC components are thought to assemble at ~35 chromatin entry sites along the X chromosome, including two of which encode the *roX* genes. The sites serve as nucleation sites for spreading the DCC complex into the flanking chromatin [145]. The *roX* RNAs play an essential role in targeting the DCC to the X chromosome, and deletion of them both results in mislocalization of DCC and acetylation of histone H4 at Lys 16 by the MOF histone acetyltransferase [146,147]. 

## 3. Heterochromatin

Heterochromatin is transcriptionally less active and associated with gene silencing, less accessible than euchromatin. Emil Heitz first coined the term ‘heterochromatin’ when preparing cytological samples of chromosomes to distinguish regions that remained strongly stained throughout the cell cycle from those that became invisible during interphase [148]. Heterochromatin spatially segregates from euchromatin in the nucleus and is localized preferentially toward the nuclear periphery and regions surrounding the nucleolus [149,150]. Due to the compacted state of the heterochromatin, Heitz hypothesized that the zones were genetically inactive, paving the way for future studies on how chromatin compaction regulates gene expression. The connection between gene silencing and heterochromatin was established when X-ray-induced chromosome rearrangements relocated a gene into the proximity of a heterochromatic region and caused variegated pigmentation of fly eyes due to gene expression inactivation without any alteration to gene expression [151,152]. Heterochromatin is categorized into facultative and constitutive heterochromatin. Facultative heterochromatin usually assembles on regions that contain developmentally regulated genes that are kept silent upon developmental cues. In contrast to this, constitutive heterochromatin occurs at the same genomic regions in every cell type and once established, it is propagated throughout the life of an individual. Unlike euchromatin, which shows characteristic nucleosome positioning at promoters due to specific DNA sequence guided DNA–protein interactions (i.e., interactions between core promoters and transcription factors), heterochromatin is largely defined by sequence independent epigenetic mechanisms and lacks nucleosome positioning.

### 3.1. Constitutive Heterochromatin

Constitutive heterochromatin is a more static structure that ensures a condensed and transcriptionally less active chromatin conformation. Constitutive heterochromatin is characterized by the presence of histone marks such as Histone 3 Lysine 9 trimethylation (H3K9me3) and occurs at highly repetitive and gene-poor regions such as pericentric regions, sub-telomeric regions and transposable elements [153,154]. H3K9me3 is imparted by histone methyltransferases (HMTs), including Suv39h in mammals, Su(var)3–9 in *Drosophila,* and Clr4 in yeast [155,156]. Classes of repetitive DNA previously thought to have no known biological function and were referred to as “junk DNA”, have appeared to play important cellular roles [157]. For example, pericentric regions consist of repetitive tandem satellite repeats and are responsible for accurate chromosome segregation by preventing premature chromatid separation [158]. Satellites underlying constitutive heterochromatin are not conserved and can greatly vary between different organisms, further suggesting that constitutive heterochromatin is controlled epigenetically. 

#### 3.1.1. Position Effect Variegation (PEV)

PEV is a phenomenon caused when a gene normally located in euchromatin undergoes abnormal juxtaposition to heterochromatic regions by genomic rearrangements is silencing in some cells [151]. PEV silencing results due to the compact packaging of the newly positioned gene in a heterochromatic environment. Multiple euchromatic genes become inactive when placed near or in heterochromatin as a consequence of heterochromatin “spreading” along the chromosome from the adjacent constitutive heterochromatin region [159]. In *Drosophila*, most rearrangements that have been shown to result in PEV map to pericentric regions and the genes involved in PEV are involved in constitutive heterochromatin formation.

#### 3.1.2. Constitutive Heterochromatin Establishment and Maintenance

**Figure 3:**
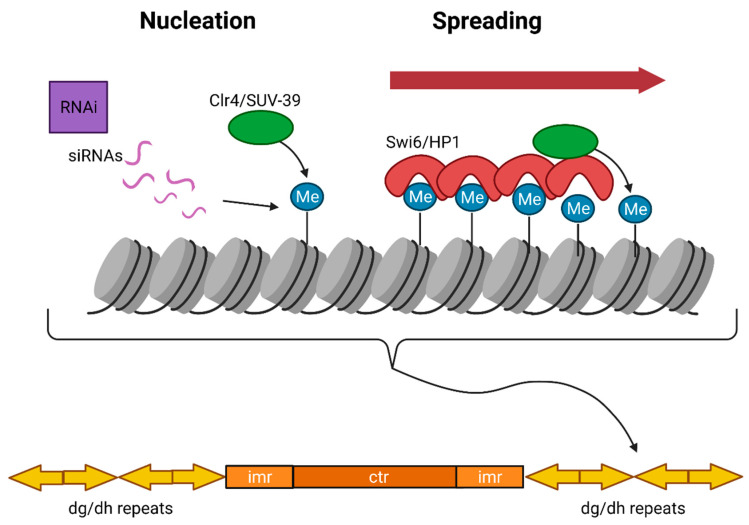
Establishment and spreading of constitutive heterochromatin in *S. pombe.* In *S. pombe*, H3K9me3 is established by the RNAi pathway in which siRNAs facilitate the recruitment of Clr4 H3K9me3 HMT. H3K9me3 is recognized by Swi6, which then recruits Clr4 to adjacent repeats leading to the spreading of constitutive heterochromatin until the process is stopped by boundary elements (not shown).

The mechanism of constitutive heterochromatin formation is well characterized in the fission yeast *S. pombe*, where the RNAi pathway plays the key role in the establishment of heterochromatin. *S. pombe* genome contains only one homolog each of the core RNAi machinery, Dicer (Dcr), Argonaute (Ago), and RNA-dependent RNA polymerase (RdRP) [160,161]. In the absence of RNAi components, reporter gene silencing near centromeres is lost and is accompanied by the accumulation of transcripts derived from heterochromatic repeats [162]. Deletion of genes coding for the RNAi machinery also results in the loss of histone H3K9 methylation, heterochromatin assembly and defects in chromosome segregation [162,163]. Pericentric regions in *S. pombe* consist of inverted repeats called *dg* and *dh* repeats that transcribe into ncRNAs. Resulting ncRNAs are processed by the Dicer ribonuclease into small interfering RNAs (siRNAs), which then recruit the RNA-induced transcriptional silencing (RITS) complex in cis onto pericentric regions [164]. The RITS complex recruits the H3K9me3 HMT, Clr4, which places H3K9me3 modification onto the pericentric chromatin. H3K9me3 modification is specifically recognized by Heterochromatin protein 1 (HP1) or Swi6 in *S. pombe* [165]. HP1 is a heterochromatin protein that binds the H3K9 methyl-marked constitutive heterochromatin and condenses reconstituted H3K9me containing nucleosome arrays in vitro [166,167]. HP1 further recruits Clr4, the main producer of the H3K9me mark, and establishes a feedback loop for heterochromatin assembly [156]. Pericentric regions are surrounded by boundary elements that prevent heterochromatin from spreading into euchromatic regions that contain their own histone modifications (Figure 3).

#### 3.1.3. Heterochromatin Compaction

The most striking feature of constitutive heterochromatin is highly compact packaging and a variety of functions impacted by heterochromatin, including heritable gene repression and maintenance of chromosome integrity, are due in part to compaction of the underlying chromatin. HP1 uses oligomerization to compact chromatin into phase-separated condensates [168,169,170,171]. The ability of HP1 to compact nucleosomal arrays in vitro is relatively lesser than the immense compaction at pericentric regions seen in vivo, suggesting that additional factors are required to condense H3K9me heterochromatin in the nucleus [167]. HP1 contains an RNA-binding domain and RNA binding is required for heterochromatin localization of mammalian HP1 to pericentric regions [172]. Studies also suggest that architectural RNAs and RNA-binding proteins contribute to H3K9me heterochromatin compaction [135,167,173,174,175]. Depletion of RNA leads to dispersion of H3K9me foci and compromises the ability of recombinant HP1 to bind to pericentric heterochromatin [172]. In mouse, specific depletion of pericentric repeat transcripts leads to a decreased number of chromocenters (clusters of pericentric regions) [173]. Similar to constitutive heterochromatin, it is also possible that chromatin–RNA bridging interactions occur within domains of facultative heterochromatin that are compacted by the PRC1 complex. The functions of PRC1 in both development and gene silencing are thought to involve lncRNAs [17,176,177]. For example, CAT7, a long non-coding RNA, has been found to tune PRC1 function during human development [177]. 

While there are many histone variants associated with heterochromatic regions, a well-characterized one is MacroH2A, a variant generally considered transcriptionally repressive and associates with the inactive X-chromosome and inactive genes [178,179]. MacroH2A is enriched on heterochromatic regions during development and ESC differentiation [180]. Another histone variant in Arabidopsis, H2A.W, was found to enhance chromatin condensation by promoting fiber–fiber interactions [27].

Recently it has been demonstrated that heterochromatin forms phase-separated liquid condensates [170,171,181]. Chromatin compaction by HP1 protein, Swi6, results in phase-separated liquid condensates. Swi6 increases the accessibility and dynamics of buried histone residues in a nucleosome, thus indicating that Swi6 couples oligomerization to the phase separation of chromatin by dynamic exposure of buried nucleosomal regions [19]. 

### 3.2. Facultative Heterochromatin

**Figure 4:**
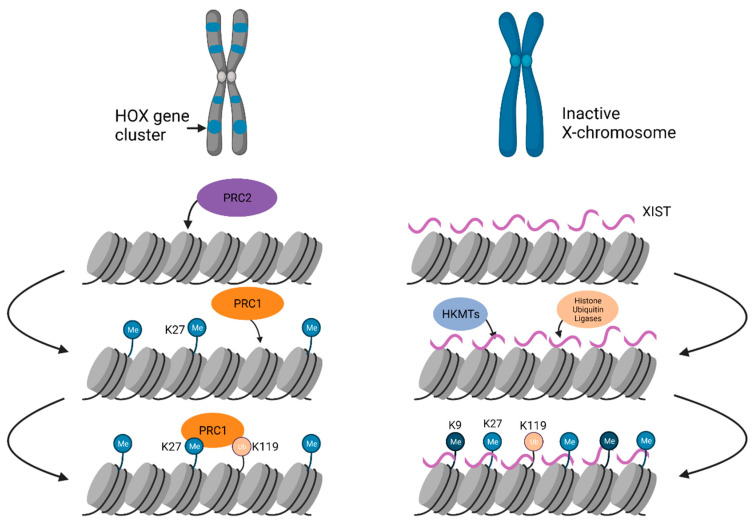
Establishment of facultative heterochromatin. Left–domain-wide facultative heterochromatin formation on developmentally regulated loci such as HOX gene clusters distributed throughout chromosomes. PRC1 deposits H3K27me, which is recognized by PRC2 that catalyzes H3K27me3 and H3K119ub formation on these domains. Right–chromosome-wide facultative heterochromatin over an entire Xi. The lncRNA Xist binds to the X-chromosome, which then recruits PcG proteins, HMTs, and histone ubiquitin ligases to deposit H3K119ub, H3K9me, and H3K27me modifications to establish compact heterochromatin.

Facultative heterochromatin is assembled on developmentally regulated loci, which can switch between transcriptionally active and inactive chromatin states during development and differentiation. As a result, not all cell types will have an identical pattern of facultative heterochromatin distribution in the genome. Facultative heterochromatin is regulated by polycomb group (PcG) proteins that impart histone 3 lysine 27 trimethylation (H3K27me3) marks. Facultative heterochromatin can be found on an entire chromosome, as seen in X-chromosome inactivation, or on discrete domains that are distributed genome-wide (Figure 4). 

#### 3.2.1. Chromosome-Wide Facultative Heterochromatin Formation on the Xi

X chromosome inactivation (XCI), is a classic example of the developmentally controlled silent facultative heterochromatin formation, allowing only one X chromosome to be expressed in female mammals. [182]. One random X chromosome is subjected to chromosome-wide chromatin condensation, which is maintained throughout the entire life of the organism. The suppression of X-linked genes depends on several chromatin modifications and *trans*-acting factors, including the lncRNA *Xist* (X-inactive specific transcript) that appears on the Xi. Xi territory occupies the perinucleolar location [183]. In the early embryo, *Xist* transcribes and spreads in *cis* on the entire Xi and recruits PcG complexes, such as Polycomb repressive complex 1 (PRC1), which catalyzes ubiquitylation of histone H2A at lysine 119 (H2AK119ub1) and PRC2 complex, which catalyzes trimethylation of H3K27me3 [184,185,186]. The coating of the Xi with repressive H3K27me3 excludes the transcriptional machinery, thereby silencing the entire Xi [187]. The formation of repressive chromatin is accompanied by the absence of histone marks associated with active genes, such as histone acetylation or H3K4me [188,189]. *Xist* uses 3D proximal contacts to spread across the entire chromosome and recruits machinery for catalyzing repressive chromatin modifications [190,191]. 

#### 3.2.2. Genome-Wide Facultative Heterochromatin Formation

Facultative heterochromatin is also present at discrete genomic loci, including autosomal imprinted genomic loci and HOX gene clusters. Facultative heterochromatin of imprinted autosomal genes is characterized by histone hypoacetylation, H3K9me2, H3K27me, and DNA methylation with the inactive alleles [192]. Notably, the H2A variant, macroH2A, was also found on imprinted autosomal genes and can serve as a marker to identify imprinted genes [193]. HOX genes are a subset of homeobox genes that specify regions of the body plan of an embryo along the head–tail axis and facultative heterochromatin controls the expression of these domains during development. In *Drosophila*, establishment and maintenance of facultative heterochromatin over HOX gene clusters via PcG machinery involves ncRNA transcribed from polycomb response elements (PREs) [194]. The PRE ncRNAs are processed by RNAi machinery into small RNAs [194]. Dicer-2, PIWI, and Argonaute1, three RNAi components colocalize with PcG proteins and the frequency of PcG-dependent chromosomal associations of endogenous homeotic genes are reduced in the mutants of these proteins suggesting that RNAi and PcG proteins mediate long-distance physical interactions [194]. PREs have been shown to control transgene expression up to 100 kb downstream, suggesting that PRE-bound PcG proteins contact distal regions [22]. A lncRNA, HOTAIR, can bring H3K27 methylated and H3K4 unmethylated sites in close proximity to one another by binding two distinct histone-modifying complexes, PRC2 and LSD1/CoREST/REST complex at their 5’ and 3’ ends, respectively [195]. The multiple zinc-finger containing architectural protein CCCTC-binding factor (CTCF), which acts as an insulator between facultative heterochromatin and euchromatin, contains an RNA-binding domain and is known to interact with *Xist* and *Tsix* [196,197,198,199]. CTCF RNA binding mutants show compromised self-association, binding to chromatin, and chromatin loop formation, suggesting that CTCF–RNA interactions regulate chromatin looping [199,200,201]. Multiple ncRNA components cooperate in *cis* or in *trans* with PcG proteins to provide context specificity to PcG protein recruitment and function in facultative heterochromatin formation [202,203,204,205]. Furthermore, many PREs form R-loops that are recognized by both PRC1 and PRC2 in vitro where they open DNA bubbles [206]. Once PRC1 and PRC2 are recruited to the target loci, they mediate the methylation of H3K27 and the monoubiquitination of H2AK119 along with chromatin compaction [207].

## 4. Centromeric Chromatin

Centromeres are the sites of spindle binding, where replicated chromatids or homologous chromosomes are pulled apart in chromosome segregation during meiosis and mitosis. While the overall chromosome segregation machinery is highly conserved across eukaryotes, the DNA and protein components of centromeric chromatin are evolving rapidly. The incompatibilities generated by evolving centromeric components may be responsible for the reproductive isolation of emerging species [208]. Centromeric sequences range from small ~100 bp point centromeres of budding yeast to several megabase long highly repetitive centromeres of plants and mammals. Centromeres of plants and mammals consist of long arrays of tandemly arranged DNA repeats called satellites. The length of satellite monomers in most plants and mammals is around nucleosomal DNA length. Rice centromeric satellites confer translational and rotational phasing on centromeric nucleosomes [209,210]. Centromeric chromatin is highly specialized and is characterized by the presence of nucleosomes in which histone H3 is replaced by its variant CENP-A. The CENP-A chromatin acts as the foundation for the assembly of a multiproteinaceous structure called the kinetochore. Dimethylation of H3K4, which is associated with “poised” euchromatin, has also been found to colocalize with CENP-A [211]. Centromeric chromatin lacks H3K9 di or trimethylation and is hypoacetylated [211].

In the absence of conserved centromeric DNA sequences, CENP-A chromatin has been considered as the epigenetic mark of centromeres [209,212,213,214]. The epigenetic nature of centromeric chromatin is supported by the occurrence of neocentromeres, ectopic functional centromeres that form on non-centromeric locations that lack native centromeric satellite sequences [215]. Neocentromeres assemble CENP-A chromatin efficiently and have been reported in various organisms [216,217,218,219,220].

### 4.1. CENP-A

Unlike histone H3, which is highly conserved across eukaryotes, CENP-A is strikingly divergent likely because H3 interacts with the entire genome while CENP-A interacts with centromeric DNA, which is rapidly evolving [221,222,223,224]. The rapid evolution of centromeric DNA and essential centromere proteins is explained by the centromeric drive model that suggests that the asymmetric meiosis in which one product of meiosis becomes the oocyte nucleus and the remaining three meiotic products are lost as polar bodies and lost, provides an opportunity for centromeric sequence variants to preferentially transmit to the next generation [208,221]. There is evidence that the asymmetry of the meiotic tetrad allows chromosomes to compete for their orientation to become the oocyte during meiosis [225,226]. To suppress the harmful effect of centromere drive in males where meiosis is symmetric, CENP-A has been proposed to act as the suppresser of centromere drive by accumulating mutations that stabilize its interactions with centromeric DNA variants [227,228,229,230]. 

Depletion of CENP-A results in mislocalization of kinetochore proteins, but depletion of kinetochore proteins does not affect CENP-A localization suggesting that CENP-A assembly acts upstream of the kinetochore formation [231,232]. When CENP-A is over-expressed, it mislocalizes to non-centromeric regions and forms ectopic kinetochores leading to chromosome segregation defects [233]. Centromeric DNA replicates mid to late S phase in *Drosophila* and human cells and early S phase in *S. pombe.* Unlike canonical H3 nucleosomes deposited during the rS phase, CENP-A nucleosome assembly is replication-independent and, in humans, occurs in late mitosis and throughout G1 [234,235,236,237,238]. Human CENP-A nucleosomes are stable and segregate to daughter chromatids during S phase, thus offering a mechanism for centromere inheritance [238]. Replenishment of CENP-A during the cell cycle is critical to centromere stability. CENP-A nucleosomes are strongly associated with other DNA-bound centromere-specific proteins, including CENP-B, CENP-C, and the CENP-TWSX histone-fold complex, to form a coherent assemblage [229,239,240]. 

### 4.2. Holliday Junction Recognition Protein

CENP-A is recruited to centromeres by a CENP-A-specific histone chaperone called Holliday Junction Recognition Protein (HJURP). HJURP stabilizes soluble CENP-A-H4 dimers before incorporation into centromeric nucleosomes [241,242,243]. The CENP-A-H4 binding region of HJURP, in the N-terminal region, is what defines a class of CENP-A assembly factors with common ancestry. This includes Scm3 in *Saccharomyces cerevisiae*, which acts as a deposition factor for the CENP-A ortholog Cse4, and the only sequence similarity of Scm3 and HJURP is that binding domain [244,245,246,247]. Human HJURP’s central domain (CD), or mid domain (HMD), has been implicated in DNA binding [248]. While HJURP was originally described as a protein that binds cruciform structures, it has been proposed that HJURP recognizes centromeric satellites through their predicted DNA structures that are enriched on satellite centromeres and neocentromeres [249]. HJURP expression is associated with colorectal cancer and has been suggested to be a potential prognostic biomarker and a novel target for drug discovery due to the higher survival rate of patients with high HJURP expression compared to those with low expression [250].

**Figure 5:**
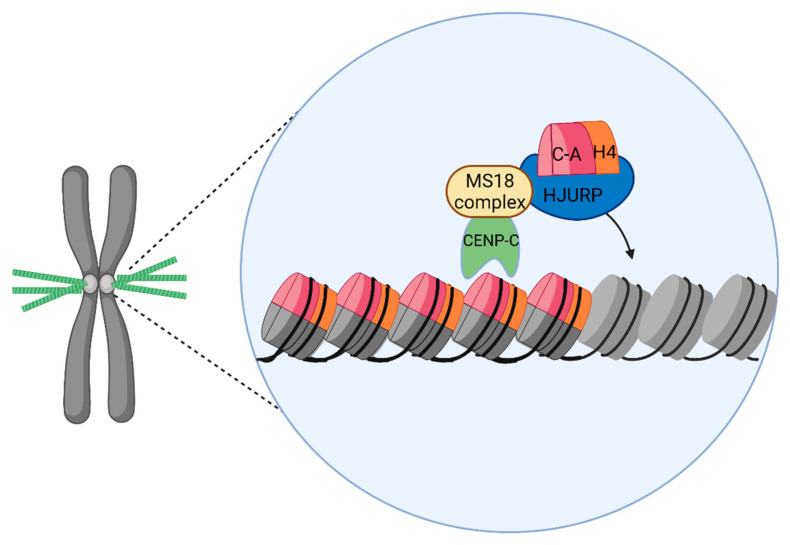
The recruitment of CENP-A to centromeres. The Mis18 complex binds to CENP-C, preceding HJURP to centromeres. HJURP then chaperones CENP-A to centromeric chromatin for deposition into nucleosomes.

Several centromeric proteins control the timing and localization of HJURP recruitment. To deposit new CENP-A to centromeres, soluble HJURP-CENP-A complex associates with the Mis18 complex, which consists of centromeric proteins Mis18α, Mis18β, and M18BP1 (Figure 5) [213,251,252,253,254,255,256,257,258,259,260]. Additionally, binding to CENP-A or CENP-C nucleosomes contributes to the centromeric recruitment of the MIS18 complex [25,254,255,256,257,258,259,260,261,262,263,264] (Figure 5).

### 4.3. CENP-A Chromatin Structure and Organization

Many approaches have been used to study centromeric chromatin, including biochemistry, biophysics, and genomics and a significant number of these studies have reported alternative CENP-A nucleosomal forms in centromeres, leading to different findings. Using elegant, functional centromeric plasmid-based genetic assays in the budding yeast, Furuyama and Henikoff demonstrated that centromeric DNA that assembles CENP-A nucleosomes undergoes positive supercoiling, which was proposed to arise due to the right-handed wrapping of centromeric DNA superhelix around CENP-A nucleosomes [265]. The positive supercoiling likely resists the stress generated on centromeric chromatin during pulling apart of sister chromatids at centromeres during anaphase. Although these findings have tremendous implications in understanding the structure of CENP-A nucleosomes, the inability to construct centromeric plasmids and extreme repetitiveness of centromeric sequences in other organisms make it difficult to further study the role of positive supercoiling in centromere function.

#### 4.3.1. Biochemistry and Biophysics-Based Findings

The widely assumed octameric nature of CENP-A nucleosomes where two copies of each of the four core histones form the nucleosome octamer was revisited when native *Drosophilia* CID arrays were found to contain all four histones in a heterotypic tetramer or “hemisome” configuration [266]. Immunoprecipitated endogenous CID particle arrays showed an average particle height half of a canonical nucleosome in atomic force microscopy (AFM) visualization. The half-height histone cores could be released from CID chromatin and were confirmed to contain CID by imaging [267]. The lack of DNA in these particles rules out the possibility that differences in the DNA wrap account for the differences in AFM height [268]. Hemisomes were also later documented in native human CENP-A arrays using AFM with recognition imaging and electron microscopy immunolabeling [269]. Subsequently, sucrose gradient purification of overexpressed FLAG-tagged CID-containing nucleosomes revealed *Drosophila* CID particles that were octamers [270]. However, the centromeric CID arrays were not confirmed to belong to centromeres and might have been derived from the misincorporation of overexpressed CID into chromosome arms. Overexpression of yeast CENP-A has been shown to misincorporate octamer-sized particles in non-centromeric locations [271,272]. More recently, AFM analysis suggests that the apparent differences in height between H3 and CENP-A nucleosomes might arise due to wrapping differences [273]. 

Although AFM is well suited to visualize the DNA and protein components of nucleosomes to probe the structures of molecular complexes at the single-molecule level both label-free and with sub-nanometer resolution, the measurements of structural parameters by AFM suffer from (1) the convolution of the molecular and AFM tip geometry, (2) the typically small sample sizes, and (3) inconsistencies associated with manual data analysis [274,275,276,277,278,279]. These problems compromise the accuracy and precision of AFM measurements and thus, care should be taken while interpreting AFM data, especially in regard to CENP-A chromatin.

#### 4.3.2. Genomics-Based Chromatin Profiling of Centromeric Chromatin

Given that CENP-A chromatin is considered to contain an epigenetic component, analysis of in vivo chromatin organization is crucial to understand the native structure of centromeric chromatin, especially in the light of conflicting evidence regarding the histone composition and stoichiometry of CENP-A nucleosomes. However, native centromeric regions have been difficult to study due to the presence of long arrays of highly homogeneous satellite repeat sequences and remain one of the last frontiers of the human genome. Although centromeric maps have been recently generated for human centromeres, utilization of these maps for functional studies remains to be attempted [280,281,282]. A bottom-up functional genomics-based approach has been applied for de novo isolation and classification of large pools of unassembled centromeric α-satellites based on CENP-A binding [227]. A large majority of CENP-A bound human functional centromeres were found to be tandem arrays of dimers [283]. Using these native α-satellite dimeric arrays as linear maps to profile CENP-A and other centromeric proteins, CENP-A was found to be tightly associated with another centromere-specific histone CENP-T and proteins CENP-B and CENP-C [229]. The CENP-A chromatin complex that results is positioned on α-satellite dimers with specific footprints, and sequence variations in α-satellite arrays are associated with changes in the CENP-A chromatin complex footprints, thus suggesting that a sequence-dependent genetic component underlies CENP-A assembly [229,230]. Additionally, these results suggest that nucleosomes at centromeric chromatin are tightly associated with other centromeric proteins and are likely more tightly packaged than those in the euchromatin. These studies did not determine the composition of CENP-A nucleosomes but based on the length of the protected DNA found in CENP-A chromatin immunoprecipitation, CENP-A nucleosomes have been proposed to be octamers by a different genomics-based study [284].

## 5. Health Implications

The regulation of chromatin is essential for controlled gene expression, development, and cell identity. Abnormal chromatin regulation can lead to pathological conditions, including cancer [285]. Chromatin deregulation is widely seen in neurodevelopmental disorders, intellectual disabilities, neurodegenerative diseases, immunodeficiency and muscle wasting syndromes [286,287,288,289,290]. Mutations in several chromatin remodeling ATPases (SWI/SNF) are associated with inherited disorders such as α-thalassemia X-linked mental retardation, B cell lymphoma, Cockayne syndrome type B and Schimke immuno-osseous dysplasia [291,292]. Altered DNA methylation in Parkinson’s disease (PD), a progressive loss of substantia nigra dopaminergic neurons and striatal projections that causes muscular rigidity and tremors, can cause increased expression of α-synuclein leading to its accumulation at sites of neuronal loss [293]. Furthermore, DNMT1 expression is reduced in PD patient brains, and DNMT1 is thought to be sequestered in the cytoplasm by α-synuclein, leading to hypo-methylated CpGs [294]. 

Epigenetic dysregulation and aberrant transcription of centromeric and heterochromatic satellite DNAs have been shown to contribute to chromosome instability, aging, and cancers [295,296,297,298,299,300,301]. Centromere and pericentromeric regions are more prone to chromosome breakage, likely due to the highly repetitive nature of the underlying DNA [302]. Human immunodeficiency-centromere instability-facial anomalies (ICF) syndrome is often diagnosed by the presence of stretched and fragile pericentromeric heterochromatin on chromosomes 1 and 16 in activated lymphocytes which results in aneuploidy and micronuclei formation [303]. Human centromeres undergo aberrant rearrangements in many tumors, causing chromosome fusions and genetic abnormalities [304,305]. Chromosome instability has been frequently observed in human tumors and has been attributed to fission events at centromeric locations [306]. Moreover, the severity of chromosome instability has been shown to closely correlate with tumor severity and poor prognosis [307].

## 6. Conclusions and Future Perspectives

Chromatin is indispensable for both packaging DNA and regulating gene expression. The core chromatin components remain mostly the same throughout the genome, but the higher-order structure and chromatin-associated regulatory proteins and protein complexes differ strikingly on different chromatin types designated to perform distinct functions. The primary outcome of these differences is a change in the accessibility of DNA to the transcription machinery leading to the activation or repression of target DNA loci. Although euchromatin forms only a small fraction of the total chromatin, it is associated with a variety of chromatin remodelers, histone modifications, histone variants and chaperones possibly to regulate the active transcription of different genes differentially and tightly. Additionally, long-range interactions between enhancers and promoters have appeared to play an important role by bringing together the core transcription machinery with chromatin-associated factors that enhance the transcription and provide tissue-specificity to the gene expression. 

Heterochromatin forms the major fraction of the total chromatin and is associated with two sets of heterochromatic proteins depending upon its constitutive and facultative nature. Due to its ability to undergo strong compaction and silence the transcription, the constitutive chromatin packages a large amount of our genome and preserves genome integrity. Therefore, constitutive heterochromatin-associated factors such as H3K9me3 modifying enzymes and HP1 homologs and ncRNAs are mainly involved in establishing the permanent compact state of their target substrate chromatin [169,308,309]. On the other hand, facultative heterochromatin is more versatile as its structure and function change during development and differentiation [191,204]. Its regulation involves a variety of chromatin modifying complexes and ncRNAs. Although recently, the focus in the field has shifted to investigating the phase separation properties of heterochromatin, it remains to be seen if phase separation provides any mechanistic contribution to the structure and function of heterochromatin [170,171,310].

Finally, centromeric chromatin is the most unique among all types of chromatin as it is dedicated to performing a single yet essential function of mediating chromosome segregation and is characterized by the presence of histone variant CENP-A, one of the least conserved histone variants. CENP-A chromatin marks the underlying genomic loci for centromere function and provides the structural platform for kinetochore formation [311]. Unlike euchromatin and heterochromatin, where the structure of the nucleosome core particle is well understood, the nucleosome composition and dimensions of CENP-A chromatin are still being investigated to get a clear understanding. With the improvement of long-read sequencing technology and chromatin profiling techniques, it is now becoming feasible to understand centromeric chromatin in its native form in vivo [229,230,281,282].

Due to their fundamental role in gene expression, mechanistic understanding of chromatin complexes is crucial for both their biological function and biomedical relevance. Chromatin factors including histone modifications, histone variants, and chromatin remodelers are important in several biological processes including differentiation, memory formation, and cell fate, and altered chromatin regulation has been implicated in many human diseases including PD, ICF, and many cancers [61,62,63,77,293,303,304,305]. 

In summary, the chromatin field has made significant progress and facilitated the understanding of both fundamental mechanisms underlying the regulation of gene expression and genome packaging and involvement of chromatin components in several diseases. 

## Figures and Tables

**Table 1:** Features of euchromatin, heterochromatin and centromeric chromatin.

	Euchromatin	Heterochromatin	Centromeric Chromatin	References
Function	Active transcription	Gene silencing, genome integrity	Chromosome segregation	[23]
Structure	Open/unfolded	Closed/condensed	Somewhat compact	[24]
Histone modifications	H3K4me3 H3K27ac H3K36me	H3K9me3 H3k27me3 H3K119ub	H3K4me2 H4K20me	[25,26]
Histone variants	H2A.Z H3.3 H2A.B	MacroH2A H2A.W	CENP-A (CenH3)	[11,27]
Chaperones	Asf1 CAF-1 HIRA NAP1 FACT	Daxx FACT	HJURP	[28,29]

## Data Availability

Not applicable.

## References

[1] Flemming W. (1882). Zellsubstanz, Kern und Zelltheilung.

[2] Avery O.T., Macleod C.M., McCarty M. (1944). Studies on the Chemical Nature of the Substance Inducing Transformation of Pneumococcal Types: Induction of Transformation by a Desoxyribonucleic Acid Fraction Isolated from Pneumococcus Type III. J. Exp. Med..

[3] Heitz E., Bauer H. (1933). Beweise für die Chromosomennatur der Kernschleifen in den Knäuelkernen von *Bibio hortulanus* L.. Z. Zellforsch. Mikrosk. Anat..

[4] Olins D.E., Olins A.L. (2003). Chromatin history: Our view from the bridge. Nat. Rev. Mol. Cell Biol..

[5] Kornberg R.D. (1974). Chromatin structure: A repeating unit of histones and DNA. Science.

[6] Bram S. (1972). The function of the structure of DNA in chromosomes. Biochimie.

[7] Nicodemi M., Pombo A. (2014). Models of chromosome structure. Curr. Opin. Cell Biol..

[8] Richards B.M., Pardon J.F., Pooley A.S. (1971). Chromatin structure. Biochem. J..

[9] Luger K., Mader A.W., Richmond R.K., Sargent D.F., Richmond T.J. (1997). Crystal structure of the nucleosome core particle at 2.8 A resolution. Nature.

[10] Ahmad K., Henikoff S. (2002). The histone variant H3.3 marks active chromatin by replication-independent nucleosome assembly. Mol. Cell.

[11] Martire S., Banaszynski L.A. (2020). The roles of histone variants in fine-tuning chromatin organization and function. Nat. Rev. Mol. Cell Biol..

[12] Zhang T., Cooper S., Brockdorff N. (2015). The interplay of histone modifications—Writers that read. EMBO Rep..

[13] Castellano-Pozo M., Santos-Pereira J.M., Rondón A.G., Barroso S., Andújar E., Pérez-Alegre M., García-Muse T., Aguilera A. (2013). R loops are linked to histone H3 S10 phosphorylation and chromatin condensation. Mol. Cell.

[14] Jørgensen S., Schotta G., Sørensen C.S. (2013). Histone H4 lysine 20 methylation: Key player in epigenetic regulation of genomic integrity. Nucleic Acids Res..

[15] Nakayama J., Rice J.C., Strahl B.D., Allis C.D., Grewal S.I. (2001). Role of histone H3 lysine 9 methylation in epigenetic control of heterochromatin assembly. Science.

[16] Palmer D.K., O’Day K., Wener M.H., Andrews B.S., Margolis R.L. (1987). A 17-kD centromere protein (CENP-A) copurifies with nucleosome core particles and with histones. J. Cell Biol..

[17] Francis N.J., Kingston R.E., Woodcock C.L. (2004). Chromatin compaction by a polycomb group protein complex. Science.

[18] Phillips J.E., Corces V.G. (2009). CTCF: Master weaver of the genome. Cell.

[19] Sanulli S., Trnka M.J., Dharmarajan V., Tibble R.W., Pascal B.D., Burlingame A.L., Griffin P.R., Gross J.D., Narlikar G.J. (2019). HP1 reshapes nucleosome core to promote phase separation of heterochromatin. Nature.

[20] Hong J., Feng H., Wang F., Ranjan A., Chen J., Jiang J., Ghirlando R., Xiao T.S., Wu C., Bai Y. (2014). The catalytic subunit of the SWR1 remodeler is a histone chaperone for the H2A.Z-H2B dimer. Mol. Cell.

[21] Schubert H.L., Wittmeyer J., Kasten M.M., Hinata K., Rawling D.C., Héroux A., Cairns B.R., Hill C.P. (2013). Structure of an actin-related subcomplex of the SWI/SNF chromatin remodeler. Proc. Natl. Acad. Sci. USA.

[22] Schwartz Y.B., Pirrotta V. (2007). Polycomb silencing mechanisms and the management of genomic programmes. Nat. Rev. Genet..

[23] Grewal S.I., Jia S. (2007). Heterochromatin revisited. Nat. Rev. Genet..

[24] Allshire R.C., Madhani H.D. (2018). Ten principles of heterochromatin formation and function. Nat. Rev. Mol. Cell Biol..

[25] Hori T., Shang W.H., Hara M., Ariyoshi M., Arimura Y., Fujita R., Kurumizaka H., Fukagawa T. (2017). Association of M18BP1/KNL2 with CENP-A Nucleosome Is Essential for Centromere Formation in Non-mammalian Vertebrates. Dev. Cell.

[26] Zhang Y., Sun Z., Jia J., Du T., Zhang N., Tang Y., Fang Y., Fang D. (2021). Overview of Histone Modification. Adv. Exp. Med. Biol..

[27] Yelagandula R., Stroud H., Holec S., Zhou K., Feng S., Zhong X., Muthurajan U.M., Nie X., Kawashima T., Groth M. (2014). The histone variant H2A.W defines heterochromatin and promotes chromatin condensation in Arabidopsis. Cell.

[28] Sun Z., Filipescu D., Andrade J., Gaspar-Maia A., Ueberheide B., Bernstein E. (2018). Transcription-associated histone pruning demarcates macroH2A chromatin domains. Nat. Struct. Mol. Biol..

[29] Hammond C.M., Strømme C.B., Huang H., Patel D.J., Groth A. (2017). Histone chaperone networks shaping chromatin function. Nat. Rev. Mol. Cell Biol..

[30] Adam M., Robert F., Larochelle M., Gaudreau L. (2001). H2A.Z is required for global chromatin integrity and for recruitment of RNA polymerase II under specific conditions. Mol. Cell. Biol..

[31] Venkatesh S., Workman J.L. (2015). Histone exchange, chromatin structure and the regulation of transcription. Nat. Rev. Mol. Cell Biol..

[32] Smale S.T., Kadonaga J.T. (2003). The RNA polymerase II core promoter. Annu. Rev. Biochem..

[33] Venkatesh S., Smolle M., Li H., Gogol M.M., Saint M., Kumar S., Natarajan K., Workman J.L. (2012). Set2 methylation of histone H3 lysine 36 suppresses histone exchange on transcribed genes. Nature.

[34] DiFiore J.V., Ptacek T.S., Wang Y., Li B., Simon J.M., Strahl B.D. (2020). Unique and Shared Roles for Histone H3K36 Methylation States in Transcription Regulation Functions. Cell Rep..

[35] Draker R., Ng M.K., Sarcinella E., Ignatchenko V., Kislinger T., Cheung P. (2012). A combination of H2A.Z and H4 acetylation recruits Brd2 to chromatin during transcriptional activation. PLoS Genet..

[36] Jin C., Zang C., Wei G., Cui K., Peng W., Zhao K., Felsenfeld G. (2009). H3.3/H2A.Z double variant-containing nucleosomes mark ‘nucleosome-free regions’ of active promoters and other regulatory regions. Nat. Genet..

[37] Radman-Livaja M., Rando O.J. (2010). Nucleosome positioning: How is it established, and why does it matter?. Dev. Biol..

[38] Struhl K., Segal E. (2013). Determinants of nucleosome positioning. Nat. Struct. Mol. Biol..

[39] Bird A.P. (1980). DNA methylation and the frequency of CpG in animal DNA. Nucleic Acids Res..

[40] Lister R., Pelizzola M., Dowen R.H., Hawkins R.D., Hon G., Tonti-Filippini J., Nery J.R., Lee L., Ye Z., Ngo Q.M. (2009). Human DNA methylomes at base resolution show widespread epigenomic differences. Nature.

[41] Jjingo D., Conley A.B., Yi S.V., Lunyak V.V., Jordan I.K. (2012). On the presence and role of human gene-body DNA methylation. Oncotarget.

[42] Maunakea A.K., Nagarajan R.P., Bilenky M., Ballinger T.J., D’Souza C., Fouse S.D., Johnson B.E., Hong C., Nielsen C., Zhao Y. (2010). Conserved role of intragenic DNA methylation in regulating alternative promoters. Nature.

[43] Neri F., Rapelli S., Krepelova A., Incarnato D., Parlato C., Basile G., Maldotti M., Anselmi F., Oliviero S. (2017). Intragenic DNA methylation prevents spurious transcription initiation. Nature.

[44] Shivaswamy S., Bhinge A., Zhao Y., Jones S., Hirst M., Iyer V.R. (2008). Dynamic remodeling of individual nucleosomes across a eukaryotic genome in response to transcriptional perturbation. PLoS Biol..

[45] Hampsey M. (1998). Molecular genetics of the RNA polymerase II general transcriptional machinery. Microbiol. Mol. Biol. Rev. MMBR.

[46] Tserel L., Kolde R., Rebane A., Kisand K., Org T., Peterson H., Vilo J., Peterson P. (2010). Genome-wide promoter analysis of histone modifications in human monocyte-derived antigen presenting cells. BMC Genom..

[47] Deaton A.M., Bird A. (2011). CpG islands and the regulation of transcription. Genes Dev..

[48] Guenther M.G., Levine S.S., Boyer L.A., Jaenisch R., Young R.A. (2007). A chromatin landmark and transcription initiation at most promoters in human cells. Cell.

[49] Hughes A.L., Kelley J.R., Klose R.J. (2020). Understanding the interplay between CpG island-associated gene promoters and H3K4 methylation. Biochim. Biophys. Acta Gene Regul. Mech..

[50] Suzuki M.M., Bird A. (2008). DNA methylation landscapes: Provocative insights from epigenomics. Nat. Rev. Genet..

[51] Hamiche A., Shuaib M. (2013). Chaperoning the histone H3 family. Biochim. Biophys. Acta.

[52] Birnbaum R.Y., Clowney E.J., Agamy O., Kim M.J., Zhao J., Yamanaka T., Pappalardo Z., Clarke S.L., Wenger A.M., Nguyen L. (2012). Coding exons function as tissue-specific enhancers of nearby genes. Genome Res..

[53] Gillies S.D., Morrison S.L., Oi V.T., Tonegawa S. (1983). A tissue-specific transcription enhancer element is located in the major intron of a rearranged immunoglobulin heavy chain gene. Cell.

[54] Core L.J., Martins A.L., Danko C.G., Waters C.T., Siepel A., Lis J.T. (2014). Analysis of nascent RNA identifies a unified architecture of initiation regions at mammalian promoters and enhancers. Nat. Genet..

[55] Dillon N., Trimborn T., Strouboulis J., Fraser P., Grosveld F. (1997). The effect of distance on long-range chromatin interactions. Mol. Cell.

[56] Tolhuis B., Palstra R.J., Splinter E., Grosveld F., de Laat W. (2002). Looping and interaction between hypersensitive sites in the active beta-globin locus. Mol. Cell.

[57] Wijgerde M., Grosveld F., Fraser P. (1995). Transcription complex stability and chromatin dynamics in vivo. Nature.

[58] Benabdallah N.S., Williamson I., Illingworth R.S., Kane L., Boyle S., Sengupta D., Grimes G.R., Therizols P., Bickmore W.A. (2019). Decreased Enhancer-Promoter Proximity Accompanying Enhancer Activation. Mol. Cell.

[59] Goldberg A.D., Banaszynski L.A., Noh K.M., Lewis P.W., Elsaesser S.J., Stadler S., Dewell S., Law M., Guo X., Li X. (2010). Distinct factors control histone variant H3.3 localization at specific genomic regions. Cell.

[60] Creyghton M.P., Cheng A.W., Welstead G.G., Kooistra T., Carey B.W., Steine E.J., Hanna J., Lodato M.A., Frampton G.M., Sharp P.A. (2010). Histone H3K27ac separates active from poised enhancers and predicts developmental state. Proc. Natl. Acad. Sci. USA.

[61] Gidekel S., Bergman Y. (2002). A unique developmental pattern of Oct-3/4 DNA methylation is controlled by a cis-demodification element. J. Biol. Chem..

[62] Horvath S. (2013). DNA methylation age of human tissues and cell types. Genome Biol..

[63] Bali P., Im H.I., Kenny P.J. (2011). Methylation, memory and addiction. Epigenetics.

[64] Santos-Rosa H., Schneider R., Bannister A.J., Sherriff J., Bernstein B.E., Emre N.C., Schreiber S.L., Mellor J., Kouzarides T. (2002). Active genes are tri-methylated at K4 of histone H3. Nature.

[65] Sims R.J., Nishioka K., Reinberg D. (2003). Histone lysine methylation: A signature for chromatin function. Trends Genet..

[66] Ng H.H., Robert F., Young R.A., Struhl K. (2003). Targeted recruitment of Set1 histone methylase by elongating Pol II provides a localized mark and memory of recent transcriptional activity. Mol. Cell.

[67] Clouaire T., Webb S., Skene P., Illingworth R., Kerr A., Andrews R., Lee J.H., Skalnik D., Bird A. (2012). Cfp1 integrates both CpG content and gene activity for accurate H3K4me3 deposition in embryonic stem cells. Genes Dev..

[68] Denissov S., Hofemeister H., Marks H., Kranz A., Ciotta G., Singh S., Anastassiadis K., Stunnenberg H.G., Stewart A.F. (2014). Mll2 is required for H3K4 trimethylation on bivalent promoters in embryonic stem cells, whereas Mll1 is redundant. Development.

[69] Murton B.L., Chin W.L., Ponting C.P., Itzhaki L.S. (2010). Characterising the binding specificities of the subunits associated with the KMT2/Set1 histone lysine methyltransferase. J. Mol. Biol..

[70] Wang Z., Song J., Milne T.A., Wang G.G., Li H., Allis C.D., Patel D.J. (2010). Pro isomerization in MLL1 PHD3-bromo cassette connects H3K4me readout to CyP33 and HDAC-mediated repression. Cell.

[71] Zhang T., Zhang Z., Dong Q., Xiong J., Zhu B. (2020). Histone H3K27 acetylation is dispensable for enhancer activity in mouse embryonic stem cells. Genome Biol..

[72] Bian C., Xu C., Ruan J., Lee K.K., Burke T.L., Tempel W., Barsyte D., Li J., Wu M., Zhou B.O. (2011). Sgf29 binds histone H3K4me2/3 and is required for SAGA complex recruitment and histone H3 acetylation. EMBO J..

[73] Gao Y., Chen L., Han Y., Wu F., Yang W.S., Zhang Z., Huo T., Zhu Y., Yu C., Kim H. (2020). Acetylation of histone H3K27 signals the transcriptional elongation for estrogen receptor alpha. Commun. Biol..

[74] Strahl B.D., Grant P.A., Briggs S.D., Sun Z.W., Bone J.R., Caldwell J.A., Mollah S., Cook R.G., Shabanowitz J., Hunt D.F. (2002). Set2 is a nucleosomal histone H3-selective methyltransferase that mediates transcriptional repression. Mol. Cell. Biol..

[75] Pokholok D.K., Harbison C.T., Levine S., Cole M., Hannett N.M., Lee T.I., Bell G.W., Walker K., Rolfe P.A., Herbolsheimer E. (2005). Genome-wide map of nucleosome acetylation and methylation in yeast. Cell.

[76] Venkatesh S., Workman J.L. (2013). Set2 mediated H3 lysine 36 methylation: Regulation of transcription elongation and implications in organismal development. Wiley Interdiscip. Rev. Dev. Biol..

[77] Buschbeck M., Hake S.B. (2017). Variants of core histones and their roles in cell fate decisions, development and cancer. Nat. Rev. Mol. Cell Biol..

[78] Mizuguchi G., Shen X., Landry J., Wu W.H., Sen S., Wu C. (2004). ATP-driven exchange of histone H2AZ variant catalyzed by SWR1 chromatin remodeling complex. Science.

[79] Willhoft O., Ghoneim M., Lin C.L., Chua E.Y.D., Wilkinson M., Chaban Y., Ayala R., McCormack E.A., Ocloo L., Rueda D.S. (2018). Structure and dynamics of the yeast SWR1-nucleosome complex. Science.

[80] Shia W.J., Li B., Workman J.L. (2006). SAS-mediated acetylation of histone H4 Lys 16 is required for H2A.Z incorporation at subtelomeric regions in *Saccharomyces cerevisiae*. Genes Dev..

[81] Bano D., Piazzesi A., Salomoni P., Nicotera P. (2017). The histone variant H3.3 claims its place in the crowded scene of epigenetics. Aging.

[82] Frank D., Doenecke D., Albig W. (2003). Differential expression of human replacement and cell cycle dependent H3 histone genes. Gene.

[83] Schwartz B.E., Ahmad K. (2005). Transcriptional activation triggers deposition and removal of the histone variant H3.3. Genes Dev..

[84] Chow C.M., Georgiou A., Szutorisz H., Maia e Silva A., Pombo A., Barahona I., Dargelos E., Canzonetta C., Dillon N. (2005). Variant histone H3.3 marks promoters of transcriptionally active genes during mammalian cell division. EMBO Rep..

[85] Ray-Gallet D., Quivy J.P., Scamps C., Martini E.M., Lipinski M., Almouzni G. (2002). HIRA is critical for a nucleosome assembly pathway independent of DNA synthesis. Mol. Cell.

[86] Lewis P.W., Elsaesser S.J., Noh K.M., Stadler S.C., Allis C.D. (2010). Daxx is an H3.3-specific histone chaperone and cooperates with ATRX in replication-independent chromatin assembly at telomeres. Proc. Natl. Acad. Sci. USA.

[87] Wong L.H., McGhie J.D., Sim M., Anderson M.A., Ahn S., Hannan R.D., George A.J., Morgan K.A., Mann J.R., Choo K.H. (2010). ATRX interacts with H3.3 in maintaining telomere structural integrity in pluripotent embryonic stem cells. Genome Res..

[88] Gehre M., Bunina D., Sidoli S., Lubke M.J., Diaz N., Trovato M., Garcia B.A., Zaugg J.B., Noh K.M. (2020). Lysine 4 of histone H3.3 is required for embryonic stem cell differentiation, histone enrichment at regulatory regions and transcription accuracy. Nat. Genet..

[89] Chadwick B.P., Willard H.F. (2001). A novel chromatin protein, distantly related to histone H2A, is largely excluded from the inactive X chromosome. J. Cell Biol..

[90] Winkler C., Steingrube D.S., Altermann W., Schlaf G., Max D., Kewitz S., Emmer A., Kornhuber M., Banning-Eichenseer U., Staege M.S. (2012). Hodgkin’s lymphoma RNA-transfected dendritic cells induce cancer/testis antigen-specific immune responses. Cancer Immunol. Immunother..

[91] Ioudinkova E.S., Barat A., Pichugin A., Markova E., Sklyar I., Pirozhkova I., Robin C., Lipinski M., Ogryzko V., Vassetzky Y.S. (2012). Distinct distribution of ectopically expressed histone variants H2A.Bbd and MacroH2A in open and closed chromatin domains. PLoS ONE.

[92] Loyola A., Bonaldi T., Roche D., Imhof A., Almouzni G. (2006). PTMs on H3 variants before chromatin assembly potentiate their final epigenetic state. Mol. Cell.

[93] Tolstorukov M.Y., Goldman J.A., Gilbert C., Ogryzko V., Kingston R.E., Park P.J. (2012). Histone variant H2A.Bbd is associated with active transcription and mRNA processing in human cells. Mol. Cell.

[94] Sansoni V., Casas-Delucchi C.S., Rajan M., Schmidt A., Bonisch C., Thomae A.W., Staege M.S., Hake S.B., Cardoso M.C., Imhof A. (2014). The histone variant H2A.Bbd is enriched at sites of DNA synthesis. Nucleic Acids Res..

[95] Molaro A., Wood A.J., Janssens D., Kindelay S.M., Eickbush M.T., Wu S., Singh P., Muller C.H., Henikoff S., Malik H.S. (2020). Biparental contributions of the H2A.B histone variant control embryonic development in mice. PLoS Biol..

[96] McKnight S.L., Miller O.L. (1977). Electron microscopic analysis of chromatin replication in the cellular blastoderm *Drosophila melanogaster* embryo. Cell.

[97] Stillman B. (1986). Chromatin assembly during SV40 DNA replication in vitro. Cell.

[98] Smith D.J., Whitehouse I. (2012). Intrinsic coupling of lagging-strand synthesis to chromatin assembly. Nature.

[99] Campos E.I., Fillingham J., Li G., Zheng H., Voigt P., Kuo W.H., Seepany H., Gao Z., Day L.A., Greenblatt J.F. (2010). The program for processing newly synthesized histones H3.1 and H4. Nat. Struct. Mol. Biol.

[100] Apta-Smith M.J., Hernandez-Fernaud J.R., Bowman A.J. (2018). Evidence for the nuclear import of histones H3.1 and H4 as monomers. EMBO J..

[101] Tagami H., Ray-Gallet D., Almouzni G., Nakatani Y. (2004). Histone H3.1 and H3.3 complexes mediate nucleosome assembly pathways dependent or independent of DNA synthesis. Cell.

[102] Tyler J.K., Adams C.R., Chen S.R., Kobayashi R., Kamakaka R.T., Kadonaga J.T. (1999). The RCAF complex mediates chromatin assembly during DNA replication and repair. Nature.

[103] Weber C.M., Ramachandran S., Henikoff S. (2014). Nucleosomes are context-specific, H2A.Z-modulated barriers to RNA polymerase. Mol. Cell.

[104] Szenker E., Ray-Gallet D., Almouzni G. (2011). The double face of the histone variant H3.3. Cell Res..

[105] Drane P., Ouararhni K., Depaux A., Shuaib M., Hamiche A. (2010). The death-associated protein DAXX is a novel histone chaperone involved in the replication-independent deposition of H3.3. Genes Dev..

[106] Mosammaparast N., Ewart C.S., Pemberton L.F. (2002). A role for nucleosome assembly protein 1 in the nuclear transport of histones H2A and H2B. EMBO J..

[107] Jamai A., Imoberdorf R.M., Strubin M. (2007). Continuous histone H2B and transcription-dependent histone H3 exchange in yeast cells outside of replication. Mol. Cell.

[108] Xu M., Long C., Chen X., Huang C., Chen S., Zhu B. (2010). Partitioning of histone H3-H4 tetramers during DNA replication-dependent chromatin assembly. Science.

[109] Andrews A.J., Chen X., Zevin A., Stargell L.A., Luger K. (2010). The histone chaperone Nap1 promotes nucleosome assembly by eliminating nonnucleosomal histone DNA interactions. Mol. Cell.

[110] Ito T., Bulger M., Pazin M.J., Kobayashi R., Kadonaga J.T. (1997). ACF, an ISWI-containing and ATP-utilizing chromatin assembly and remodeling factor. Cell.

[111] Torigoe S.E., Urwin D.L., Ishii H., Smith D.E., Kadonaga J.T. (2011). Identification of a rapidly formed nonnucleosomal histone-DNA intermediate that is converted into chromatin by ACF. Mol. Cell.

[112] Hondele M., Stuwe T., Hassler M., Halbach F., Bowman A., Zhang E.T., Nijmeijer B., Kotthoff C., Rybin V., Amlacher S. (2013). Structural basis of histone H2A-H2B recognition by the essential chaperone FACT. Nature.

[113] Kemble D.J., McCullough L.L., Whitby F.G., Formosa T., Hill C.P. (2015). FACT Disrupts Nucleosome Structure by Binding H2A-H2B with Conserved Peptide Motifs. Mol. Cell.

[114] Valieva M.E., Armeev G.A., Kudryashova K.S., Gerasimova N.S., Shaytan A.K., Kulaeva O.I., McCullough L.L., Formosa T., Georgiev P.G., Kirpichnikov M.P. (2016). Large-scale ATP-independent nucleosome unfolding by a histone chaperone. Nat. Struct. Mol. Biol..

[115] Rippe K., Schrader A., Riede P., Strohner R., Lehmann E., Längst G. (2007). DNA sequence- and conformation-directed positioning of nucleosomes by chromatin-remodeling complexes. Proc. Natl. Acad. Sci. USA.

[116] Tyagi M., Imam N., Verma K., Patel A.K. (2016). Chromatin remodelers: We are the drivers!!. Nucleus.

[117] Marmorstein R., Berger S.L. (2001). Structure and function of bromodomains in chromatin-regulating complexes. Gene.

[118] Armstrong J.A., Emerson B.M. (1998). Transcription of chromatin: These are complex times. Curr. Opin. Genet. Dev..

[119] Clapier C.R., Iwasa J., Cairns B.R., Peterson C.L. (2017). Mechanisms of action and regulation of ATP-dependent chromatin-remodelling complexes. Nat. Rev. Mol. Cell Biol..

[120] Corona D.F., Tamkun J.W. (2004). Multiple roles for ISWI in transcription, chromosome organization and DNA replication. Biochim. Biophys. Acta.

[121] Boyer L.A., Latek R.R., Peterson C.L. (2004). The SANT domain: A unique histone-tail-binding module?. Nat. Rev. Mol. Cell Biol..

[122] Dang W., Bartholomew B. (2007). Domain architecture of the catalytic subunit in the ISW2-nucleosome complex. Mol. Cell. Biol..

[123] Grüne T., Brzeski J., Eberharter A., Clapier C.R., Corona D.F., Becker P.B., Müller C.W. (2003). Crystal structure and functional analysis of a nucleosome recognition module of the remodeling factor ISWI. Mol. Cell.

[124] Hochheimer A., Zhou S., Zheng S., Holmes M.C., Tjian R. (2002). TRF2 associates with DREF and directs promoter-selective gene expression in *Drosophila*. Nature.

[125] Xiao H., Sandaltzopoulos R., Wang H.M., Hamiche A., Ranallo R., Lee K.M., Fu D., Wu C. (2001). Dual functions of largest NURF subunit NURF301 in nucleosome sliding and transcription factor interactions. Mol. Cell.

[126] Kunert N., Brehm A. (2009). Novel Mi-2 related ATP-dependent chromatin remodelers. Epigenetics.

[127] Tran H.G., Steger D.J., Iyer V.R., Johnson A.D. (2000). The chromo domain protein chd1p from budding yeast is an ATP-dependent chromatin-modifying factor. EMBO J..

[128] Denslow S.A., Wade P.A. (2007). The human Mi-2/NuRD complex and gene regulation. Oncogene.

[129] Konev A.Y., Tribus M., Park S.Y., Podhraski V., Lim C.Y., Emelyanov A.V., Vershilova E., Pirrotta V., Kadonaga J.T., Lusser A. (2007). CHD1 motor protein is required for deposition of histone variant H3.3 into chromatin in vivo. Science.

[130] Allen H.F., Wade P.A., Kutateladze T.G. (2013). The NuRD architecture. Cell. Mol. Life Sci. CMLS.

[131] Szerlong H., Hinata K., Viswanathan R., Erdjument-Bromage H., Tempst P., Cairns B.R. (2008). The HSA domain binds nuclear actin-related proteins to regulate chromatin-remodeling ATPases. Nat. Struct. Mol. Biol..

[132] Papamichos-Chronakis M., Watanabe S., Rando O.J., Peterson C.L. (2011). Global regulation of H2A.Z localization by the INO80 chromatin-remodeling enzyme is essential for genome integrity. Cell.

[133] Pradhan S.K., Su T., Yen L., Jacquet K., Huang C., Côté J., Kurdistani S.K., Carey M.F. (2016). EP400 Deposits H3.3 into Promoters and Enhancers during Gene Activation. Mol. Cell.

[134] Maison C., Bailly D., Peters A.H., Quivy J.P., Roche D., Taddei A., Lachner M., Jenuwein T., Almouzni G. (2002). Higher-order structure in pericentric heterochromatin involves a distinct pattern of histone modification and an RNA component. Nat. Genet..

[135] Thakur J., Henikoff S. (2020). Architectural RNA in chromatin organization. Biochem. Soc. Trans..

[136] Bynum J.W., Volkin E. (1980). Chromatin-associated RNA: Differential extraction and characterization. Biochim. Biophys. Acta.

[137] Holmes D.S., Mayfield J.E., Sander G., Bonner J. (1972). Chromosomal RNA: Its properties. Science.

[138] Cai Z., Cao C., Ji L., Ye R., Wang D., Xia C., Wang S., Du Z., Hu N., Yu X. (2020). RIC-seq for global in situ profiling of RNA-RNA spatial interactions. Nature.

[139] Lai F., Orom U.A., Cesaroni M., Beringer M., Taatjes D.J., Blobel G.A., Shiekhattar R. (2013). Activating RNAs associate with Mediator to enhance chromatin architecture and transcription. Nature.

[140] Wang K.C., Yang Y.W., Liu B., Sanyal A., Corces-Zimmerman R., Chen Y., Lajoie B.R., Protacio A., Flynn R.A., Gupta R.A. (2011). A long noncoding RNA maintains active chromatin to coordinate homeotic gene expression. Nature.

[141] West J.A., Davis C.P., Sunwoo H., Simon M.D., Sadreyev R.I., Wang P.I., Tolstorukov M.Y., Kingston R.E. (2014). The long noncoding RNAs NEAT1 and MALAT1 bind active chromatin sites. Mol. Cell.

[142] Hou Y., Zhang R., Sun X. (2019). Enhancer LncRNAs Influence Chromatin Interactions in Different Ways. Front. Genet..

[143] Li W., Notani D., Ma Q., Tanasa B., Nunez E., Chen A.Y., Merkurjev D., Zhang J., Ohgi K., Song X. (2013). Functional roles of enhancer RNAs for oestrogen-dependent transcriptional activation. Nature.

[144] Gilfillan G.D., Dahlsveen I.K., Becker P.B. (2004). Lifting a chromosome: Dosage compensation in *Drosophila melanogaster*. FEBS Lett..

[145] Kelley R.L., Kuroda M.I. (2000). The role of chromosomal RNAs in marking the X for dosage compensation. Curr. Opin. Genet. Dev..

[146] Gu W., Szauter P., Lucchesi J.C. (1998). Targeting of MOF, a putative histone acetyl transferase, to the X chromosome of *Drosophila melanogaster*. Dev. Genet..

[147] Meller V.H., Rattner B.P. (2002). The roX genes encode redundant male-specific lethal transcripts required for targeting of the MSL complex. EMBO J..

[148] Heitz E. (1928). Das Heterochromatin der Moose.

[149] Kosak S.T., Skok J.A., Medina K.L., Riblet R., Le Beau M.M., Fisher A.G., Singh H. (2002). Subnuclear compartmentalization of immunoglobulin loci during lymphocyte development. Science.

[150] Lieberman-Aiden E., van Berkum N.L., Williams L., Imakaev M., Ragoczy T., Telling A., Amit I., Lajoie B.R., Sabo P.J., Dorschner M.O. (2009). Comprehensive mapping of long-range interactions reveals folding principles of the human genome. Science.

[151] Muller H.J. (1930). Types of visible variations induced by X-rays in *Drosophila*. J. Genet..

[152] Schultz J. (1936). Variegation in *Drosophila* and the Inert Chromosome Regions. Proc. Natl. Acad. Sci. USA.

[153] Britten R.J., Kohne D.E. (1968). Repeated sequences in DNA. Hundreds of thousands of copies of DNA sequences have been incorporated into the genomes of higher organisms. Science.

[154] McClintock C.B. (1951). Chromosome organization and genic expression. Cold Spring Harb. Symp. Quant. Biol..

[155] Peters A.H., O’Carroll D., Scherthan H., Mechtler K., Sauer S., Schofer C., Weipoltshammer K., Pagani M., Lachner M., Kohlmaier A. (2001). Loss of the Suv39h histone methyltransferases impairs mammalian heterochromatin and genome stability. Cell.

[156] Rea S., Eisenhaber F., O’Carroll D., Strahl B.D., Sun Z.W., Schmid M., Opravil S., Mechtler K., Ponting C.P., Allis C.D. (2000). Regulation of chromatin structure by site-specific histone H3 methyltransferases. Nature.

[157] Ohno S. (1972). So much “junk” DNA in our genome. Brookhaven Symp. Biol..

[158] Bernard P., Maure J.F., Partridge J.F., Genier S., Javerzat J.P., Allshire R.C. (2001). Requirement of heterochromatin for cohesion at centromeres. Science.

[159] Baker W.K. (1968). Position-effect variegation. Adv. Genet..

[160] Aravind L., Watanabe H., Lipman D.J., Koonin E.V. (2000). Lineage-specific loss and divergence of functionally linked genes in eukaryotes. Proc. Natl. Acad. Sci. USA.

[161] Carmell M.A., Xuan Z., Zhang M.Q., Hannon G.J. (2002). The Argonaute family: Tentacles that reach into RNAi, developmental control, stem cell maintenance, and tumorigenesis. Genes Dev..

[162] Volpe T.A., Kidner C., Hall I.M., Teng G., Grewal S.I., Martienssen R.A. (2002). Regulation of heterochromatic silencing and histone H3 lysine-9 methylation by RNAi. Science.

[163] Volpe T., Schramke V., Hamilton G.L., White S.A., Teng G., Martienssen R.A., Allshire R.C. (2003). RNA interference is required for normal centromere function in fission yeast. Chromosome Res..

[164] Martienssen R.A., Zaratiegui M., Goto D.B. (2005). RNA interference and heterochromatin in the fission yeast *Schizosaccharomyces pombe*. Trends Genet..

[165] Lachner M., O’Carroll D., Rea S., Mechtler K., Jenuwein T. (2001). Methylation of histone H3 lysine 9 creates a binding site for HP1 proteins. Nature.

[166] Almouzni G., Probst A.V. (2011). Heterochromatin maintenance and establishment: Lessons from the mouse pericentromere. Nucleus.

[167] Azzaz A.M., Vitalini M.W., Thomas A.S., Price J.P., Blacketer M.J., Cryderman D.E., Zirbel L.N., Woodcock C.L., Elcock A.H., Wallrath L.L. (2014). Human heterochromatin protein 1α promotes nucleosome associations that drive chromatin condensation. J. Biol. Chem..

[168] Canzio D., Chang E.Y., Shankar S., Kuchenbecker K.M., Simon M.D., Madhani H.D., Narlikar G.J., Al-Sady B. (2011). Chromodomain-mediated oligomerization of HP1 suggests a nucleosome-bridging mechanism for heterochromatin assembly. Mol. Cell.

[169] Canzio D., Liao M., Naber N., Pate E., Larson A., Wu S., Marina D.B., Garcia J.F., Madhani H.D., Cooke R. (2013). A conformational switch in HP1 releases auto-inhibition to drive heterochromatin assembly. Nature.

[170] Larson A.G., Elnatan D., Keenen M.M., Trnka M.J., Johnston J.B., Burlingame A.L., Agard D.A., Redding S., Narlikar G.J. (2017). Liquid droplet formation by HP1α suggests a role for phase separation in heterochromatin. Nature.

[171] Strom A.R., Emelyanov A.V., Mir M., Fyodorov D.V., Darzacq X., Karpen G.H. (2017). Phase separation drives heterochromatin domain formation. Nature.

[172] Muchardt C., Guilleme M., Seeler J.S., Trouche D., Dejean A., Yaniv M. (2002). Coordinated methyl and RNA binding is required for heterochromatin localization of mammalian HP1alpha. EMBO Rep..

[173] Huo X., Ji L., Zhang Y., Lv P., Cao X., Wang Q., Yan Z., Dong S., Du D., Zhang F. (2020). The Nuclear Matrix Protein SAFB Cooperates with Major Satellite RNAs to Stabilize Heterochromatin Architecture Partially through Phase Separation. Mol. Cell.

[174] Machida S., Takizawa Y., Ishimaru M., Sugita Y., Sekine S., Nakayama J.I., Wolf M., Kurumizaka H. (2018). Structural Basis of Heterochromatin Formation by Human HP1. Mol. Cell.

[175] Thakur J., Fang H., Llagas T., Disteche C.M., Henikoff S. (2019). Architectural RNA is required for heterochromatin organization. bioRxiv.

[176] Boettiger A.N., Bintu B., Moffitt J.R., Wang S., Beliveau B.J., Fudenberg G., Imakaev M., Mirny L.A., Wu C.T., Zhuang X. (2016). Super-resolution imaging reveals distinct chromatin folding for different epigenetic states. Nature.

[177] Ray M.K., Wiskow O., King M.J., Ismail N., Ergun A., Wang Y., Plys A.J., Davis C.P., Kathrein K., Sadreyev R. (2016). CAT7 and cat7l Long Non-coding RNAs Tune Polycomb Repressive Complex 1 Function during Human and Zebrafish Development. J. Biol. Chem..

[178] Costanzi C., Stein P., Worrad D.M., Schultz R.M., Pehrson J.R. (2000). Histone macroH2A1 is concentrated in the inactive X chromosome of female preimplantation mouse embryos. Development.

[179] Gamble M.J., Frizzell K.M., Yang C., Krishnakumar R., Kraus W.L. (2010). The histone variant macroH2A1 marks repressed autosomal chromatin, but protects a subset of its target genes from silencing. Genes Dev..

[180] Pehrson J.R., Costanzi C., Dharia C. (1997). Developmental and tissue expression patterns of histone macroH2A1 subtypes. J. Cell Biochem..

[181] Plys A.J., Davis C.P., Kim J., Rizki G., Keenen M.M., Marr S.K., Kingston R.E. (2019). Phase separation of Polycomb-repressive complex 1 is governed by a charged disordered region of CBX2. Genes Dev..

[182] Wutz A. (2011). Gene silencing in X-chromosome inactivation: Advances in understanding facultative heterochromatin formation. Nat. Rev. Genet..

[183] Zhang L.F., Huynh K.D., Lee J.T. (2007). Perinucleolar targeting of the inactive X during S phase: Evidence for a role in the maintenance of silencing. Cell.

[184] De Napoles M., Mermoud J.E., Wakao R., Tang Y.A., Endoh M., Appanah R., Nesterova T.B., Silva J., Otte A.P., Vidal M. (2004). Polycomb group proteins Ring1A/B link ubiquitylation of histone H2A to heritable gene silencing and X inactivation. Dev. Cell.

[185] Gendrel A.V., Heard E. (2014). Noncoding RNAs and epigenetic mechanisms during X-chromosome inactivation. Annu. Rev. Cell Dev. Biol..

[186] Plath K., Fang J., Mlynarczyk-Evans S.K., Cao R., Worringer K.A., Wang H., de la Cruz C.C., Otte A.P., Panning B., Zhang Y. (2003). Role of histone H3 lysine 27 methylation in X inactivation. Science.

[187] Chaumeil J., Le Baccon P., Wutz A., Heard E. (2006). A novel role for Xist RNA in the formation of a repressive nuclear compartment into which genes are recruited when silenced. Genes Dev..

[188] Heard E., Rougeulle C., Arnaud D., Avner P., Allis C.D., Spector D.L. (2001). Methylation of histone H3 at Lys-9 is an early mark on the X chromosome during X inactivation. Cell.

[189] Okamoto I., Otte A.P., Allis C.D., Reinberg D., Heard E. (2004). Epigenetic dynamics of imprinted X inactivation during early mouse development. Science.

[190] Engreitz J.M., Pandya-Jones A., McDonel P., Shishkin A., Sirokman K., Surka C., Kadri S., Xing J., Goren A., Lander E.S. (2013). The Xist lncRNA exploits three-dimensional genome architecture to spread across the X chromosome. Science.

[191] Simon M.D., Pinter S.F., Fang R., Sarma K., Rutenberg-Schoenberg M., Bowman S.K., Kesner B.A., Maier V.K., Kingston R.E., Lee J.T. (2013). High-resolution Xist binding maps reveal two-step spreading during X-chromosome inactivation. Nature.

[192] Feil R., Berger F. (2007). Convergent evolution of genomic imprinting in plants and mammals. Trends Genet..

[193] Choo J.H., Kim J.D., Chung J.H., Stubbs L., Kim J. (2006). Allele-specific deposition of macroH2A1 in imprinting control regions. Hum. Mol. Genet..

[194] Grimaud C., Bantignies F., Pal-Bhadra M., Ghana P., Bhadra U., Cavalli G. (2006). RNAi components are required for nuclear clustering of Polycomb group response elements. Cell.

[195] Tsai M.C., Manor O., Wan Y., Mosammaparast N., Wang J.K., Lan F., Shi Y., Segal E., Chang H.Y. (2010). Long noncoding RNA as modular scaffold of histone modification complexes. Science.

[196] Narendra V., Rocha P.P., An D., Raviram R., Skok J.A., Mazzoni E.O., Reinberg D. (2015). CTCF establishes discrete functional chromatin domains at the Hox clusters during differentiation. Science.

[197] Ong C.T., Corces V.G. (2014). CTCF: An architectural protein bridging genome topology and function. Nat. Rev. Genet..

[198] Kung J.T., Kesner B., An J.Y., Ahn J.Y., Cifuentes-Rojas C., Colognori D., Jeon Y., Szanto A., del Rosario B.C., Pinter S.F. (2015). Locus-specific targeting to the X chromosome revealed by the RNA interactome of CTCF. Mol. Cell.

[199] Saldana-Meyer R., Gonzalez-Buendia E., Guerrero G., Narendra V., Bonasio R., Recillas-Targa F., Reinberg D. (2014). CTCF regulates the human p53 gene through direct interaction with its natural antisense transcript, Wrap53. Genes Dev..

[200] Hansen A.S., Hsieh T.S., Cattoglio C., Pustova I., Saldana-Meyer R., Reinberg D., Darzacq X., Tjian R. (2019). Distinct Classes of Chromatin Loops Revealed by Deletion of an RNA-Binding Region in CTCF. Mol. Cell.

[201] Saldana-Meyer R., Rodriguez-Hernaez J., Escobar T., Nishana M., Jacome-Lopez K., Nora E.P., Bruneau B.G., Tsirigos A., Furlan-Magaril M., Skok J. (2019). RNA Interactions Are Essential for CTCF-Mediated Genome Organization. Mol. Cell.

[202] Mainguy G., Koster J., Woltering J., Jansen H., Durston A. (2007). Extensive polycistronism and antisense transcription in the mammalian Hox clusters. PLoS ONE.

[203] Petruk S., Sedkov Y., Riley K.M., Hodgson J., Schweisguth F., Hirose S., Jaynes J.B., Brock H.W., Mazo A. (2006). Transcription of bxd noncoding RNAs promoted by trithorax represses Ubx in cis by transcriptional interference. Cell.

[204] Rinn J.L., Kertesz M., Wang J.K., Squazzo S.L., Xu X., Brugmann S.A., Goodnough L.H., Helms J.A., Farnham P.J., Segal E. (2007). Functional demarcation of active and silent chromatin domains in human HOX loci by noncoding RNAs. Cell.

[205] Davidovich C., Cech T.R. (2015). The recruitment of chromatin modifiers by long noncoding RNAs: Lessons from PRC2. RNA.

[206] Alecki C., Chiwara V., Sanz L.A., Grau D., Arias Pérez O., Boulier E.L., Armache K.J., Chédin F., Francis N.J. (2020). RNA-DNA strand exchange by the *Drosophila* Polycomb complex PRC2. Nat. Commun..

[207] Vire E., Brenner C., Deplus R., Blanchon L., Fraga M., Didelot C., Morey L., Van Eynde A., Bernard D., Vanderwinden J.M. (2006). The Polycomb group protein EZH2 directly controls DNA methylation. Nature.

[208] Henikoff S., Ahmad K., Malik H.S. (2001). The centromere paradox: Stable inheritance with rapidly evolving DNA. Science.

[209] Melters D.P., Bradnam K.R., Young H.A., Telis N., May M.R., Ruby J.G., Sebra R., Peluso P., Eid J., Rank D. (2013). Comparative analysis of tandem repeats from hundreds of species reveals unique insights into centromere evolution. Genome Biol..

[210] Zhang T., Talbert P.B., Zhang W., Wu Y., Yang Z., Henikoff J.G., Henikoff S., Jiang J. (2013). The CentO satellite confers translational and rotational phasing on cenH3 nucleosomes in rice centromeres. Proc. Natl. Acad. Sci. USA.

[211] Sullivan B.A., Karpen G.H. (2004). Centromeric chromatin exhibits a histone modification pattern that is distinct from both euchromatin and heterochromatin. Nat. Struct. Mol. Biol..

[212] Mendiburo M.J., Padeken J., Fülöp S., Schepers A., Heun P. (2011). *Drosophila* CENH3 is sufficient for centromere formation. Science.

[213] Barnhart M.C., Kuich P.H., Stellfox M.E., Ward J.A., Bassett E.A., Black B.E., Foltz D.R. (2011). HJURP is a CENP-A chromatin assembly factor sufficient to form a functional de novo kinetochore. J. Cell Biol..

[214] Guse A., Carroll C.W., Moree B., Fuller C.J., Straight A.F. (2011). In vitro centromere and kinetochore assembly on defined chromatin templates. Nature.

[215] Scott K.C., Sullivan B.A. (2014). Neocentromeres: A place for everything and everything in its place. Trends Genet..

[216] Alonso A., Fritz B., Hasson D., Abrusan G., Cheung F., Yoda K., Radlwimmer B., Ladurner A.G., Warburton P.E. (2007). Co-localization of CENP-C and CENP-H to discontinuous domains of CENP-A chromatin at human neocentromeres. Genome Biol..

[217] Warburton P.E. (2004). Chromosomal dynamics of human neocentromere formation. Chromosome Res..

[218] Choo K.H. (2001). Domain organization at the centromere and neocentromere. Dev. Cell.

[219] Lo A.W., Magliano D.J., Sibson M.C., Kalitsis P., Craig J.M., Choo K.H. (2001). A novel chromatin immunoprecipitation and array (CIA) analysis identifies a 460-kb CENP-A-binding neocentromere DNA. Genome Res..

[220] Williams B.C., Murphy T.D., Goldberg M.L., Karpen G.H. (1998). Neocentromere activity of structurally acentric mini-chromosomes in *Drosophila*. Nat. Genet..

[221] Malik H.S., Henikoff S. (2001). Adaptive evolution of Cid, a centromere-specific histone in *Drosophila*. Genetics.

[222] Csink A.K., Henikoff S. (1998). Something from nothing: The evolution and utility of satellite repeats. Trends Genet..

[223] Murphy T.D., Karpen G.H. (1998). Centromeres take flight: Alpha satellite and the quest for the human centromere. Cell.

[224] Haaf T., Willard H.F. (1997). Chromosome-specific alpha-satellite DNA from the centromere of chimpanzee chromosome 4. Chromosoma.

[225] Zwick M.E., Salstrom J.L., Langley C.H. (1999). Genetic variation in rates of nondisjunction: Association of two naturally occurring polymorphisms in the chromokinesin nod with increased rates of nondisjunction in *Drosophila melanogaster*. Genetics.

[226] Pardo-Manuel de Villena F., Sapienza C. (2001). Transmission ratio distortion in offspring of heterozygous female carriers of Robertsonian translocations. Hum. Genet..

[227] Henikoff J.G., Thakur J., Kasinathan S., Henikoff S. (2015). A unique chromatin complex occupies young α-satellite arrays of human centromeres. Sci. Adv..

[228] Henikoff S., Thakur J., Kasinathan S., Talbert P.B. (2017). Remarkable Evolutionary Plasticity of Centromeric Chromatin. Cold Spring Harb. Symp. Quant. Biol..

[229] Thakur J., Henikoff S. (2018). Unexpected conformational variations of the human centromeric chromatin complex. Genes Dev..

[230] Thakur J., Henikoff S. (2016). CENPT bridges adjacent CENPA nucleosomes on young human α-satellite dimers. Genome Res..

[231] Blower M.D., Karpen G.H. (2001). The role of *Drosophila* CID in kinetochore formation, cell-cycle progression and heterochromatin interactions. Nat. Cell Biol..

[232] Takahashi K., Chen E.S., Yanagida M. (2000). Requirement of Mis6 centromere connector for localizing a CENP-A-like protein in fission yeast. Science.

[233] Heun P., Erhardt S., Blower M.D., Weiss S., Skora A.D., Karpen G.H. (2006). Mislocalization of the *Drosophila* centromere-specific histone CID promotes formation of functional ectopic kinetochores. Dev. Cell.

[234] Sogo J.M., Stahl H., Koller T., Knippers R. (1986). Structure of replicating simian virus 40 minichromosomes. The replication fork, core histone segregation and terminal structures. J. Mol. Biol..

[235] Shelby R.D., Monier K., Sullivan K.F. (2000). Chromatin assembly at kinetochores is uncoupled from DNA replication. J. Cell Biol..

[236] Sullivan B., Karpen G. (2001). Centromere identity in *Drosophila* is not determined in vivo by replication timing. J. Cell Biol..

[237] Kim S.M., Dubey D.D., Huberman J.A. (2003). Early-replicating heterochromatin. Genes Dev..

[238] Jansen L.E., Black B.E., Foltz D.R., Cleveland D.W. (2007). Propagation of centromeric chromatin requires exit from mitosis. J. Cell Biol..

[239] Kato H., Jiang J., Zhou B.R., Rozendaal M., Feng H., Ghirlando R., Xiao T.S., Straight A.F., Bai Y. (2013). A conserved mechanism for centromeric nucleosome recognition by centromere protein CENP-C. Science.

[240] Ando S., Yang H., Nozaki N., Okazaki T., Yoda K. (2002). CENP-A, -B, and -C chromatin complex that contains the I-type alpha-satellite array constitutes the prekinetochore in HeLa cells. Mol. Cell. Biol..

[241] Shuaib M., Ouararhni K., Dimitrov S., Hamiche A. (2010). HJURP binds CENP-A via a highly conserved N-terminal domain and mediates its deposition at centromeres. Proc. Natl. Acad. Sci. USA.

[242] Dunleavy E.M., Roche D., Tagami H., Lacoste N., Ray-Gallet D., Nakamura Y., Daigo Y., Nakatani Y., Almouzni-Pettinotti G. (2009). HJURP is a cell-cycle-dependent maintenance and deposition factor of CENP-A at centromeres. Cell.

[243] Foltz D.R., Jansen L.E., Bailey A.O., Yates J.R., Bassett E.A., Wood S., Black B.E., Cleveland D.W. (2009). Centromere-specific assembly of CENP-a nucleosomes is mediated by HJURP. Cell.

[244] Mizuguchi G., Xiao H., Wisniewski J., Smith M.M., Wu C. (2007). Nonhistone Scm3 and histones CenH3-H4 assemble the core of centromere-specific nucleosomes. Cell.

[245] Camahort R., Li B., Florens L., Swanson S.K., Washburn M.P., Gerton J.L. (2007). Scm3 is essential to recruit the histone h3 variant cse4 to centromeres and to maintain a functional kinetochore. Mol. Cell.

[246] Stoler S., Rogers K., Weitze S., Morey L., Fitzgerald-Hayes M., Baker R.E. (2007). Scm3, an essential *Saccharomyces cerevisiae* centromere protein required for G2/M progression and Cse4 localization. Proc. Natl. Acad. Sci. USA.

[247] Sanchez-Pulido L., Pidoux A.L., Ponting C.P., Allshire R.C. (2009). Common ancestry of the CENP-A chaperones Scm3 and HJURP. Cell.

[248] Müller S., Montes de Oca R., Lacoste N., Dingli F., Loew D., Almouzni G. (2014). Phosphorylation and DNA binding of HJURP determine its centromeric recruitment and function in CenH3(CENP-A) loading. Cell Rep..

[249] Kasinathan S., Henikoff S. (2018). Non-B-Form DNA Is Enriched at Centromeres. Mol. Biol. Evol..

[250] Kang D.H., Woo J., Kim H., Kim S.Y., Ji S., Jaygal G., Ahn T.S., Kim H.J., Kwak H.J., Kim C.J. (2020). Prognostic Relevance of HJURP Expression in Patients with Surgically Resected Colorectal Cancer. Int. J. Mol. Sci..

[251] Pan D., Walstein K., Take A., Bier D., Kaiser N., Musacchio A. (2019). Mechanism of centromere recruitment of the CENP-A chaperone HJURP and its implications for centromere licensing. Nat. Commun..

[252] Fujita Y., Hayashi T., Kiyomitsu T., Toyoda Y., Kokubu A., Obuse C., Yanagida M. (2007). Priming of centromere for CENP-A recruitment by human hMis18alpha, hMis18beta, and M18BP1. Dev. Cell.

[253] Bernad R., Sánchez P., Rivera T., Rodríguez-Corsino M., Boyarchuk E., Vassias I., Ray-Gallet D., Arnaoutov A., Dasso M., Almouzni G. (2011). Xenopus HJURP and condensin II are required for CENP-A assembly. J. Cell Biol..

[254] French B.T., Westhorpe F.G., Limouse C., Straight A.F. (2017). Xenopus laevis M18BP1 Directly Binds Existing CENP-A Nucleosomes to Promote Centromeric Chromatin Assembly. Dev. Cell.

[255] Shono N., Ohzeki J., Otake K., Martins N.M., Nagase T., Kimura H., Larionov V., Earnshaw W.C., Masumoto H. (2015). CENP-C and CENP-I are key connecting factors for kinetochore and CENP-A assembly. J. Cell Sci..

[256] Dambacher S., Deng W., Hahn M., Sadic D., Fröhlich J., Nuber A., Hoischen C., Diekmann S., Leonhardt H., Schotta G. (2012). CENP-C facilitates the recruitment of M18BP1 to centromeric chromatin. Nucleus.

[257] Moree B., Meyer C.B., Fuller C.J., Straight A.F. (2011). CENP-C recruits M18BP1 to centromeres to promote CENP-A chromatin assembly. J. Cell Biol..

[258] Stellfox M.E., Nardi I.K., Knippler C.M., Foltz D.R. (2016). Differential Binding Partners of the Mis18α/β YIPPEE Domains Regulate Mis18 Complex Recruitment to Centromeres. Cell Rep..

[259] Tachiwana H., Müller S., Blümer J., Klare K., Musacchio A., Almouzni G. (2015). HJURP involvement in de novo CenH3(CENP-A) and CENP-C recruitment. Cell Rep..

[260] Perpelescu M., Hori T., Toyoda A., Misu S., Monma N., Ikeo K., Obuse C., Fujiyama A., Fukagawa T. (2015). HJURP is involved in the expansion of centromeric chromatin. Mol. Biol. Cell.

[261] Logsdon G.A., Barrey E.J., Bassett E.A., DeNizio J.E., Guo L.Y., Panchenko T., Dawicki-McKenna J.M., Heun P., Black B.E. (2015). Both tails and the centromere targeting domain of CENP-A are required for centromere establishment. J. Cell Biol..

[262] Sandmann M., Talbert P., Demidov D., Kuhlmann M., Rutten T., Conrad U., Lermontova I. (2017). Targeting of Arabidopsis KNL2 to Centromeres Depends on the Conserved CENPC-k Motif in Its C Terminus. Plant Cell.

[263] Kral L. (2015). Possible identification of CENP-C in fish and the presence of the CENP-C motif in M18BP1 of vertebrates. F1000Research.

[264] Westhorpe F.G., Fuller C.J., Straight A.F. (2015). A cell-free CENP-A assembly system defines the chromatin requirements for centromere maintenance. J. Cell Biol..

[265] Furuyama T., Henikoff S. (2009). Centromeric nucleosomes induce positive DNA supercoils. Cell.

[266] Dalal Y., Wang H., Lindsay S., Henikoff S. (2007). Tetrameric structure of centromeric nucleosomes in interphase *Drosophila* cells. PLoS Biol..

[267] Wang H., Dalal Y., Henikoff S., Lindsay S. (2008). Single-epitope recognition imaging of native chromatin. Epigenetics Chromatin.

[268] Black B.E., Cleveland D.W. (2011). Epigenetic centromere propagation and the nature of CENP-a nucleosomes. Cell.

[269] Dimitriadis E.K., Weber C., Gill R.K., Diekmann S., Dalal Y. (2010). Tetrameric organization of vertebrate centromeric nucleosomes. Proc. Natl. Acad. Sci. USA.

[270] Zhang W., Colmenares S.U., Karpen G.H. (2012). Assembly of *Drosophila* centromeric nucleosomes requires CID dimerization. Mol. Cell.

[271] Qin J.Y., Zhang L., Clift K.L., Hulur I., Xiang A.P., Ren B.Z., Lahn B.T. (2010). Systematic comparison of constitutive promoters and the doxycycline-inducible promoter. PLoS ONE.

[272] Krassovsky K., Henikoff J.G., Henikoff S. (2012). Tripartite organization of centromeric chromatin in budding yeast. Proc. Natl. Acad. Sci. USA.

[273] Konrad S.F., Vanderlinden W., Frederickx W., Brouns T., Menze B.H., De Feyter S., Lipfert J. (2021). High-throughput AFM analysis reveals unwrapping pathways of H3 and CENP-A nucleosomes. Nanoscale.

[274] Bintu L., Kopaczynska M., Hodges C., Lubkowska L., Kashlev M., Bustamante C. (2011). The elongation rate of RNA polymerase determines the fate of transcribed nucleosomes. Nat. Struct. Mol. Biol..

[275] Katan A.J., Vlijm R., Lusser A., Dekker C. (2015). Dynamics of nucleosomal structures measured by high-speed atomic force microscopy. Small.

[276] Lyubchenko Y.L., Shlyakhtenko L.S. (2016). Imaging of DNA and Protein-DNA Complexes with Atomic Force Microscopy. Crit Rev. Eukaryot. Gene Expr..

[277] Miyagi A., Ando T., Lyubchenko Y.L. (2011). Dynamics of nucleosomes assessed with time-lapse high-speed atomic force microscopy. Biochemistry.

[278] Ordu O., Lusser A., Dekker N.H. (2016). Recent insights from in vitro single-molecule studies into nucleosome structure and dynamics. Biophys. Rev..

[279] Shlyakhtenko L.S., Lushnikov A.Y., Lyubchenko Y.L. (2009). Dynamics of nucleosomes revealed by time-lapse atomic force microscopy. Biochemistry.

[280] Logsdon G.A., Vollger M.R., Hsieh P., Mao Y., Liskovykh M.A., Koren S., Nurk S., Mercuri L., Dishuck P.C., Rhie A. (2021). The structure, function and evolution of a complete human chromosome 8. Nature.

[281] Miga K.H., Koren S., Rhie A., Vollger M.R., Gershman A., Bzikadze A., Brooks S., Howe E., Porubsky D., Logsdon G.A. (2020). Telomere-to-telomere assembly of a complete human X chromosome. Nature.

[282] Nurk S., Koren S., Rhie A., Rautiainen M., Bzikadze A.V., Mikheenko A., Vollger M.R., Altemose N., Uralsky L., Gershman A. (2021). The complete sequence of a human genome. bioRxiv.

[283] Alexandrov I., Kazakov A., Tumeneva I., Shepelev V., Yurov Y. (2001). Alpha-satellite DNA of primates: Old and new families. Chromosoma.

[284] Hasson D., Panchenko T., Salimian K.J., Salman M.U., Sekulic N., Alonso A., Warburton P.E., Black B.E. (2013). The octamer is the major form of CENP-A nucleosomes at human centromeres. Nat. Struct. Mol. Biol..

[285] Vardabasso C., Hasson D., Ratnakumar K., Chung C.Y., Duarte L.F., Bernstein E. (2014). Histone variants: Emerging players in cancer biology. Cell. Mol. Life Sci. CMLS.

[286] Mirabella A.C., Foster B.M., Bartke T. (2016). Chromatin deregulation in disease. Chromosoma.

[287] Lu H., Liu X., Deng Y., Qing H. (2013). DNA methylation, a hand behind neurodegenerative diseases. Front. Aging Neurosci..

[288] Kramer J.M., van Bokhoven H. (2009). Genetic and epigenetic defects in mental retardation. Int. J. Biochem. Cell Biol..

[289] Gibbons R.J., McDowell T.L., Raman S., O’Rourke D.M., Garrick D., Ayyub H., Higgs D.R. (2000). Mutations in ATRX, encoding a SWI/SNF-like protein, cause diverse changes in the pattern of DNA methylation. Nat. Genet..

[290] Jin P., Warren S.T. (2000). Understanding the molecular basis of fragile X syndrome. Hum. Mol. Genet..

[291] Huang C., Sloan E.A., Boerkoel C.F. (2003). Chromatin remodeling and human disease. Curr. Opin. Genet. Dev..

[292] Kokavec J., Podskocova J., Zavadil J., Stopka T. (2008). Chromatin remodeling and SWI/SNF2 factors in human disease. Front. Biosci. J. Virtual Libr..

[293] Jowaed A., Schmitt I., Kaut O., Wüllner U. (2010). Methylation regulates alpha-synuclein expression and is decreased in Parkinson’s disease patients’ brains. J. Neurosci. Off. J. Soc. Neurosci..

[294] Desplats P., Spencer B., Coffee E., Patel P., Michael S., Patrick C., Adame A., Rockenstein E., Masliah E. (2011). Alpha-synuclein sequesters Dnmt1 from the nucleus: A novel mechanism for epigenetic alterations in Lewy body diseases. J. Biol. Chem..

[295] Scaffidi P., Misteli T. (2006). Lamin A-dependent nuclear defects in human aging. Science.

[296] Leung W., Shaffer C.D., Reed L.K., Smith S.T., Barshop W., Dirkes W., Dothager M., Lee P., Wong J., Xiong D. (2015). *Drosophila* Muller F elements maintain a distinct set of genomic properties over 40 million years of evolution. G3 Genes Genomes Genet..

[297] Shumaker D.K., Dechat T., Kohlmaier A., Adam S.A., Bozovsky M.R., Erdos M.R., Eriksson M., Goldman A.E., Khuon S., Collins F.S. (2006). Mutant nuclear lamin A leads to progressive alterations of epigenetic control in premature aging. Proc. Natl. Acad. Sci. USA.

[298] Ehrlich M. (2009). DNA hypomethylation in cancer cells. Epigenomics.

[299] Eymery A., Callanan M., Vourc’h C. (2009). The secret message of heterochromatin: New insights into the mechanisms and function of centromeric and pericentric repeat sequence transcription. Int. J. Dev. Biol..

[300] Ting D.T., Lipson D., Paul S., Brannigan B.W., Akhavanfard S., Coffman E.J., Contino G., Deshpande V., Iafrate A.J., Letovsky S. (2011). Aberrant overexpression of satellite repeats in pancreatic and other epithelial cancers. Science.

[301] Zhu Q., Pao G.M., Huynh A.M., Suh H., Tonnu N., Nederlof P.M., Gage F.H., Verma I.M. (2011). BRCA1 tumour suppression occurs via heterochromatin-mediated silencing. Nature.

[302] Knutsen T., Gobu V., Knaus R., Padilla-Nash H., Augustus M., Strausberg R.L., Kirsch I.R., Sirotkin K., Ried T. (2005). The interactive online SKY/M-FISH & CGH database and the Entrez cancer chromosomes search database: Linkage of chromosomal aberrations with the genome sequence. Genes Chromosomes Cancer.

[303] Hagleitner M.M., Lankester A., Maraschio P., Hultén M., Fryns J.P., Schuetz C., Gimelli G., Davies E.G., Gennery A., Belohradsky B.H. (2008). Clinical spectrum of immunodeficiency, centromeric instability and facial dysmorphism (ICF syndrome). J. Med. Genet..

[304] Mitelman F., Mertens F., Johansson B. (1997). A breakpoint map of recurrent chromosomal rearrangements in human neoplasia. Nat. Genet..

[305] Padilla-Nash H.M., Heselmeyer-Haddad K., Wangsa D., Zhang H., Ghadimi B.M., Macville M., Augustus M., Schröck E., Hilgenfeld E., Ried T. (2001). Jumping translocations are common in solid tumor cell lines and result in recurrent fusions of whole chromosome arms. Genes Chromosomes Cancer.

[306] Martínez A.C., van Wely K.H. (2011). Centromere fission, not telomere erosion, triggers chromosomal instability in human carcinomas. Carcinogenesis.

[307] Ricke R.M., van Deursen J.M. (2013). Aneuploidy in health, disease, and aging. J. Cell Biol..

[308] Alekseyenko A.A., Gorchakov A.A., Zee B.M., Fuchs S.M., Kharchenko P.V., Kuroda M.I. (2014). Heterochromatin-associated interactions of *Drosophila* HP1a with dADD1, HIPP1, and repetitive RNAs. Genes Dev..

[309] Cam H.P., Sugiyama T., Chen E.S., Chen X., FitzGerald P.C., Grewal S.I. (2005). Comprehensive analysis of heterochromatin- and RNAi-mediated epigenetic control of the fission yeast genome. Nat. Genet..

[310] Guillén-Boixet J., Kopach A., Holehouse A.S., Wittmann S., Jahnel M., Schlüßler R., Kim K., Trussina I.R.E.A., Wang J., Mateju D. (2020). RNA-Induced Conformational Switching and Clustering of G3BP Drive Stress Granule Assembly by Condensation. Cell.

[311] Thakur J., Packiaraj J., Henikoff S. (2021). Sequence, Chromatin and Evolution of Satellite DNA. Int. J. Mol. Sci..

